# Rumination and Self-Compassion Moderate Mindfulness-Based Cognitive Therapy for Patients With Recurrent and Persistent Major Depressive Disorder: A Controlled Trial

**DOI:** 10.1155/da/3511703

**Published:** 2024-11-25

**Authors:** Jelle Lubbers, Dirk E. M. Geurts, Philip Spinhoven, Mira B. Cladder-Micus, Demi Ennen, Anne E. M. Speckens, Jan Spijker

**Affiliations:** ^1^Department of Psychiatry, Radboudumc Centre for Mindfulness, Nijmegen PO Box 9101, 6500, HB, Netherlands; ^2^Depression Expertise Centre, Pro Persona Mental Health Care, Nijmeegsebaan 61, Nijmegen 6525 , DX, Netherlands; ^3^Behavioural Science Institute, Radboud University Nijmegen, Nijmegen P.O. Box 9104, 6500, HE, Netherlands; ^4^Donders Institute for Brain, Cognition and Behaviour, Radboud University Nijmegen, Nijmegen P.O. Box 9010, 6500, GL, Netherlands; ^5^Institute of Psychology, Leiden University and Department of Psychiatry, Leiden University Medical Center, Wassenaarseweg 52, Leiden 2333, AK, Netherlands

**Keywords:** depressive rumination, major depressive disorder, mediator, mindfulness skills, moderator, perseverative thinking, self-compassion, working mechanisms

## Abstract

**Background:** Mindfulness-based cognitive therapy (MBCT) is effective in reducing depressive symptoms in patients with major depressive disorder (MDD). Understanding for whom and how MBCT works may allow for improvements in treatment allocation and effectiveness. In this study, our aim was to investigate depressive rumination, content-independent perseverative thinking, mindfulness skills, and self-compassion as potential moderators and mediators of MBCT.

**Methods:** In this non-randomized controlled trial, patients with persistent (*n* = 53) or recurrent MDD with (*n* = 31) or without (*n =* 51) a current depressive episode were assigned to an intervention (MBCT plus treatment as usual [TAU], *n* = 94) or control group (TAU only, *n* = 40) based on the time between the date of inclusion and the start of MBCT. Assessments were carried out before, halfway, and after 8 weeks of MBCT + TAU or TAU. Latent growth models were employed to examine moderation, while cross-lagged structural equation models were used to assess the mediating effects of several possible mediators of MBCT-induced change in depressive symptoms and overall functional impairment.

**Results:** MBCT + TAU was more effective in reducing depressive symptoms (and overall functional impairment than TAU with a medium [*d* = −0.54] and small [*d* = 0.44] effect size, respectively). Higher baseline levels of rumination and perseverative thinking and lower levels of self-compassion moderated the effect of MBCT on depressive symptoms and overall functional impairment. Task-based negative intrusive thoughts moderated the effects of MBCT on overall functional impairment. No mediators were established, particularly due to a lack of effect of MBCT on all assessed mediators at mid-treatment. For interpretative purposes, a sample split (based on Johnson–Newman values) showed moderate-to-large effects in depressive symptom reduction for those with high rumination, high perseverative thinking, and low self-compassion, while negative-to-small nonsignificant effects were found for the opposite traits.

**Conclusion:** In the future, MBCT allocation based on levels of rumination and self-compassion might lead to a more efficient reduction in depressive symptoms. Directions for mediation analysis within the context of MBCT for depression are discussed.

**Preregistration:** This study was initially preregistered in the Dutch National Trial Register (NL7842). However, due to the NTR no longer being available since June 2022, the trial was reregistered at ClinicalTrials.gov (NCT05802966, dd 09-Apr-2023). The statistical analysis plan was adjusted after the start of the trial but before the finalization of data collection (NCT05802966; ClinicalTrials.gov).

## 1. Introduction

Major depressive disorder (MDD) is a leading cause of disability worldwide and contributes considerably to the overall global burden of disease [1, 2]. Mindfulness-based cognitive therapy (MBCT) has been proven effective in preventing relapse and recurrence in MDD [3, 4]. Multiple clinical trials in recent years have also provided strong evidence for the efficacy of MBCT in reducing current depressive symptoms [5]. In addition to the evidence from controlled trials, evidence from clinical settings shows that MBCT is also effective in reducing depressive symptoms within routine clinical practice [6–8].

Despite the well-documented beneficial effects of MBCT for improving current depressive symptoms, there are considerable individual differences, and the exact working mechanisms of MBCT are far from clear [9–11]. A better understanding of individual differences in treatment effect by identification of moderators (i.e., variables that influence the strength or direction of the treatment effect) might allow for improved treatment allocation. In addition, elucidating the mechanisms by which MBCT sorts its clinical effects (i.e., mediators) is of crucial importance to even further optimize the effectiveness of MBCT.

One of the factors that plays a central role in MDD is repetitive negative thinking (RNT) [12], such as depressive rumination, which has been defined as the process of thinking perseveratively about one's feelings and problems and their possible causes and consequences [13]. Patients with current and remitted MDD ruminate more than healthy controls, and severity of rumination has been shown to be related to severity of depression [14, 15].

During MBCT, patients learn to become more aware of negative thoughts and feelings and cultivate a more accepting and self-compassionate attitude toward them. By becoming aware of automatic maladaptive cognitive processes such as depressive rumination and by learning to decenter and disengage from them, it is thought that patients break the vicious cycle of ruminative thinking that could aggravate symptoms of depression [16]. Considerable evidence shows that MBCT reduces depressive rumination in MDD [17, 18] and that those reductions are associated with reductions in depressive symptoms [9–11]. This suggests that MBCT may be particularly beneficial for patients with higher levels of rumination. Indeed, previous research indicates that baseline severity of rumination moderate the effect of MBCT on the reduction of depressive symptoms in patients with chronic (treatment-resistant) or recurrent MDD [19, 20]. Specifically, patients with treatment-resistant depression who ruminated more before treatment showed a larger decrease in depressive symptoms with MBCT compared to treatment as usual (TAU) [19]. In addition, a study combining three datasets from randomized controlled trials (RCTs) on the effectiveness of MBCT for chronic (treatment-resistant) or recurrent MDD (including Cladder-Micus et al. [19]) found MBCT to be most effective for patients with earlier onset and higher baseline levels of depressive rumination [20]. However, research on moderators of MBCT, especially baseline levels of continuous measures of constructs potentially involved in the workings of MBCT, such as rumination, and RNT [10], remains limited and needs further exploration.

In light of this, two other factors that may moderate the effects of MBCT are mindfulness and self-compassion skills. Mindfulness is defined by Kabat-Zinn [21] as “the awareness that arises from paying attention, on purpose, in the present moment and non-judgmentally.” Closely related is self-compassion, which involves approaching one's own experiences and emotions with kindness and without judgment, while recognizing that suffering and failure are part of the shared human experience [22]. Preliminary evidence indicates that these skills play a crucial role in mindfulness-based interventions (MBIs): improvements in mindfulness and self-compassion have been consistently associated with reductions in depressive symptoms [9–11]. Therefore, similar to depressive rumination, it would be valuable to assess whether levels of mindfulness skills and self-compassion before treatment would moderate MBCT's effects on depressive symptom reduction.

In addition to moderators of treatment effect, identification of mediators is important for a better understanding of the mechanisms by which MBCT sorts its clinical effects. However, the quality of studies aiming to assess mediation is problematic. Nearly all previous studies that assessed the mechanisms of MBI lack assessments during the intervention and therefore prevent conclusions on whether change in putative mediator precedes change in outcome during the intervention [9–11], which is necessary to establish “real” mediation [23]. In the context of MBCT for treatment-resistant depression, only Eisendrath et al. [24] assessed mediators and outcomes during the intervention and found no evidence that posttreatment improvement in depression severity was mediated by a differential effect of treatment on mediators at mid-treatment. However, it is important to note that their RCT primarily focused on treatment effectiveness rather than underlying mechanisms and exclusively included patients with treatment-resistant depression.

Another methodological drawback of previous studies assessing the workings of MBCT is that they often only included self-report questionnaires that may increase the risk of recall bias. To shed light on the potential mediating role of depressive rumination, and other potential mediators such as mindfulness skills and self-compassion [9–11], well-powered and more rigorously designed studies that include multiple time points (e.g., assessments before, mid-way, and after treatment) and a combination of self-report and task-based measures are required [10, 11]. In addition, more sophisticated statistical methods such as (random-intercept) cross-lagged structural equation (RI-CLSEM) panel models [25], are required because they offer a more comprehensive understanding of the dynamic interplay between change in mediator and outcome. These models account for how mediator and outcome of interest evolve over time by incorporating autoregressive (stability of a variable over time) and cross-lagged effects (how one variable at a specific time point influences another variable at a later time point), thus providing a nuanced picture of the temporal relationships. Furthermore, RI-CLSEM models allow for the analysis of individual participants, distinguishing within-person processes from stable between-person differences [25]. Especially these within-person processes are key in studying mediation.

Within the current controlled trial, our main aim was to assess the moderating and mediating role of RNT, mindfulness skills, and self-compassion in the MBCT-induced effects on depressive symptoms and overall functional impairment in a sample of patients with recurrent or persistent MDD. Our objectives were to (i) replicate the well-established beneficial effects of MBCT on (depressive) symptom reduction [5]: i.e., whether MBCT + TAU was more effective in reducing depressive symptoms (and overall functional impairment as secondary outcome) compared to TAU in the current controlled trial and to assess (ii) whether this hypothesized effect was moderated by baseline levels of RNT, mindfulness skills, and self-compassion (iii) whether a change in RNT, mindfulness skills, and self-compassion at mid-treatment mediates the MBCT-induced reduction in depressive symptoms and overall functional impairment at post-treatment.

## 2. Methods

### 2.1. Design

A controlled trial was conducted in which patients with recurrent or persistent MDD, as defined by the Diagnostic and Statistical Manual of Mental Disorders-5 (DSM-5), were assigned to one of two groups based on the time between baseline clinical assessment and predefined start of the MBCT group (5 times/year). Patients with persistent or recurrent MDD, both in remission and currently experiencing depression (Table 1), were included because the latest depression guidelines (e.g., NICE guidelines in the United Kingdom and the recently updated Dutch FMS guideline: Spijker et al., [26]) recommend MBCT as a psychological treatment option for both relapse prevention and the treatment of current depressive symptoms. When the time between clinical assessment and predefined start date of MBCT was more than 8 weeks, patients were assigned to the control group in which patients received TAU. When it was less than 8 weeks, they were assigned to the intervention group (MBCT + TAU). In both groups, assessments were conducted before, halfway, and after MBCT or waiting period. After their waiting period, participants in the control group participated in MBCT and completed assessments again halfway through and after the training. Because assessments were conducted before, halfway, and after MBCT or waiting period, our design specifically allowed us to assess whether change in hypothesized mediators precedes subsequent change in outcome.

### 2.2. Participants

**Figure 1:**
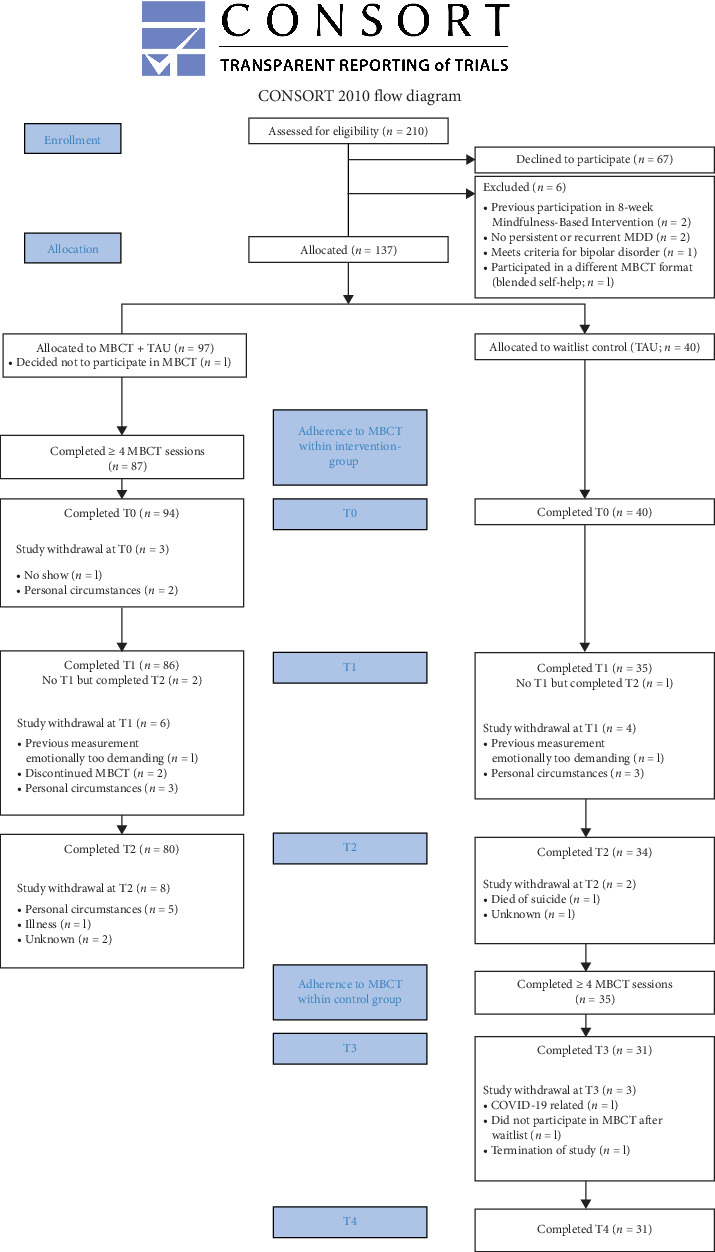
Consort flow diagram of controlled trial.

Patients (*n* = 210) with MDD who were referred for MBCT at the Radboud University Medical Center for Mindfulness or at various locations of a local mental health institute (Pro Persona) were assessed for eligibility for this study (for flow diagram, see Figure 1). To assess eligibility, patients followed routine clinical procedures of the individual institutions and underwent screenings for persistent or recurrent depression, along with other in and exclusion criteria. Depression was considered persistent if patients met the DSM-5 criteria for a “persistent depressive disorder,” which is characterized by a depressed mood for most of the day for the majority of days over at least a 2-year period. It was considered recurrent if patients met the DSM-5 criteria for “major depressive disorder” and had either at least one previous and a current episode or two past episodes of depression. Of 210 assessed patients, 137 were assigned to one of the groups of whom 134 completed a baseline assessment (Figure 1).

Inclusion criteria were (a) age ≥18; (b) diagnosis of persistent MDD or recurrent MDD (both current and remitted) according to DSM-5 criteria; and (c) ability to give informed consent.

Exclusion criteria were (a) remission of the first (not persistent) episode or a current first (not persistent) episode; (b) insufficient comprehension of the Dutch language; (c) physical, cognitive, or intellectual impairments interfering with participation; (d) formerly participated in MBCT or MBSR or another 8-week MBI; (e) bipolar disorder, schizophrenia, schizophreniform disorder, schizoaffective disorder or anorexia nervosa, or current psychosis; (f) high level of suicidality; and (g) drug (except smoking) or alcohol addiction in the past 6 months.

### 2.3. Procedures

Patients with MDD referred to the Radboudumc Expertise Centre for Mindfulness for MBCT were screened by a psychologist or psychiatrist (in training) for in and exclusion criteria during a routine clinical assessment. Patients with MDD referred for MBCT at Pro Persona mental health locations were informed about the study by a therapist during a “treatment plan interview” for MBCT. During this interview, suitability for MBCT was assessed and patients were briefly checked for in and exclusion criteria. If interested, an (video-call) appointment was made with the researcher to check eligibility more extensively using the Mini International Neuropsychiatric Interview (MINI-PLUS). The researchers were trained to administer the MINI-PLUS for screening purposes.

At both sites, when eligible, oral, and written information about the study was provided. Within a week, patients were contacted by a researcher, and appointments for baseline assessments were planned. Because of the coronavirus disease 2019 (COVID-19) pandemic, patients were given a choice to either participate in assessments in person at the Radboudumc Expertise Centre for Mindfulness (when local regulations at the time allowed this) or online via video conferencing. Patients from Pro Persona all participated in online assessments (due to practical reasons: COVID-19 pandemic and no researchers available at all Pro Persona). Informed consent was obtained during the first face-to-face appointment prior to the start of the assessment or via post-letter correspondence in case patients participated online. Physical assessments consisted of several self-report questionnaires and an experimental test battery consisting of the breathing focus task (BFT), pavlovian to instrumental transfer task (PIT), and an affective working memory update/ignore task (WMUIET). The PIT and WMUIET will be reported elsewhere. In case assessments consisted exclusively of online measurements, only the self-report questionnaires and the BFT were administered. In some instances, patients only completed the self-report questionnaires and did not perform the BFT for personal (too time consuming or energy demanding) or practical reasons (planning, technical issues with video calling).

In Radboudumc, demographic and clinical variables were obtained during routine clinical assessment. When required, missing information was extracted from the electronic patient health record. In Pro Persona, those variables were obtained during the first online video appointment with the researcher.

### 2.4. Intervention

MBCT courses were offered in accordance with the MBCT manual designed for preventing relapse in MDD patients [27]. Thus, the program comprised 8-weekly sessions lasting 2.5 h each, a 6-h silent day, and additionally included daily home practice (~45 min). The MBCT sessions were provided by certified MBCT teachers with professional background as psychologist (*n* = 5), psychomotor therapist (*n* = 3), or nurse specialist (*n* = 1). Those certified teachers (*n* = 9, years of experience as certified teacher: *Med* (P25–P75) = 6 (1–7)) met the advanced criteria of the Association of Mindfulness Based Teachers in the Netherlands and Flanders (Belgium), which are in concordance with the Good Practice guidelines of the UK Network of Mindfulness-Based Teacher Trainers [28]. After their waiting period, patients in the control group also received MBCT.

In the Netherlands, TAU may consist of psychotherapy (including but not limited to cognitive behavioral therapy or interpersonal therapy), medication, or a combination of both. It is usually delivered by a psychiatrist, psychologist, nurse specialist, general practitioner, or general practice nurse) or by a general practitioner only. Table 1 displays the distribution of current treatments at the time of the clinical assessment (prior to the study's start). TAU was not monitored during the study period.

### 2.5. Measures

#### 2.5.1. Inventory of Depressive Symptomatology-Self-Report (IDS-SR)

The IDS-SR is a 30-item self-report questionnaire that assesses the severity of depressive symptoms with good psychometric properties [29] (Dutch translation: [30]). The IDS-SR measures symptoms on a 4-point Likert scale (0–3). Severity of depressive symptoms can be categorized as no (0–13), mild (14–25), moderate (26–38), severe (39–48), or very severe (49–84) depression. Internal consistency in the current sample was good (*α* = 0.87).

#### 2.5.2. Outcome Questionnaire-45 (OQ-45)

The OQ-45 is a self-report measure of psychological and general functioning and is commonly used to assess the effect of treatment because it is sensitive to change over short periods of time [31]. In the current study, the Dutch OQ-45 [32] total score was used (ranging from 0 to 180). This total score is based on three subscales with items measuring symptom distress (56%), interpersonal relations (24%), and social role (20%). Because a higher score means lower mental health and worse functioning, we will refer to the OQ-45 as a measure of overall functional impairment. The 45 items of the OQ-45 measure symptoms on a 5-point Likert scale (0–4). De Jong and Spinhoven [32] reported excellent internal consistency for the total score in different samples (*α* ≥ 0.91), which was comparable to the current sample (*α* = 0.91).

#### 2.5.3. Brooding Subscale of the Ruminative Response Scale (RRS)—Extended Version

The RRS is a self-report questionnaire that measures levels of depressive rumination and was originally developed by Nolen-Hoeksema and Morrow [33]. Subsequent research showed that 12 of the 22 items of the RRS had too much overlap with depressive symptoms, and subsequent confirmatory factor analysis of the remaining 10 items revealed two distinctive factors: “reflection” and “brooding” [34] of which the latter is more maladaptive and more strongly related to depressive symptoms. The current study used the translated Dutch version of the RRS that contains 26-items from which the “brooding subscale” (5 items, ranging from 0 to 20) was calculated [35]. For ease of readability, we will refer to the RRS brooding subscale score as “rumination” for the remainder of this paper. Internal consistency in the current sample (*α* = 0.75) was comparable to the adequate consistency (*α* = 0.77) previously reported by Treynor et al. [34].

#### 2.5.4. Perseverative Thinking Questionnaire (PTQ)

The PTQ is a 15-item self-report questionnaire that assesses the general tendency to engage in RNT independent of thought content [36]. The PTQ measures levels of RNT on a 5-point Likert scale (0–4). The total score (ranging from 0 to 60) was used in the current study. Previously reported excellent internal consistency (*α* ≥ 0.93) [36] was comparable (*α =* 0.93) to the current sample.

#### 2.5.5. Five Facet Mindfulness Questionnaire-Short Form (FFMQ-SF)

The FFMQ-SF [37] was employed to assess mindfulness skills across the five domains: “observing,” “describing,” “acting with awareness,” “non-judgment of inner experience,” and “non-reactivity to inner experience.” The questionnaire employs a 5-point Likert scale (1–5). Because subscales have unequal numbers of items, subscales were calculated by determining the mean of corresponding item scores. Prior to calculating the mean scores, scores on negatively-phrased items were reversed. The total score was used in the current study and was calculated by summing the subscale (mean) scores. The individual subscales in the current sample at baseline demonstrated good internal consistency (*α* ranging from 0.79 to 0.85).

#### 2.5.6. Self-Compassion Scale-Short Form (SCS-SF)

The SCS assesses the levels of self-compassion in six domains: “self-kindness”, “self-judgment”, “common humanity”, “isolation”, “mindfulness,” and “overidentification” [22]. Within the current study, the 12-item short-form version [38] was used to assess those domains on a 7-point Likert scale (1–7). The total score was calculated by averaging the 12 (reversed) individual items. The total score of the 12-item SCS-SF demonstrated adequate internal consistency in two Dutch student samples and one US student sample (*α* ≥ 0.86), which was comparable to the current study (*α* = 0.86).

#### 2.5.7. BFT

The BFT was originally devised by Borkovec et al. [39] and adapted by several researchers [40–43] to assess negative intrusive thoughts, such as worry or rumination, during task performance. Because intrusive thoughts measured by the BFT are not necessarily ruminative or worrying in nature, we recently chose to define it as a measure of negative, positive, and neutral intrusive thoughts [44] and will refer to it in that manner in the remainder of this paper.

Typically the BFT consists of three phases: an initial assessment phase, followed by a phase to induce negative mood or worry, and finally a second assessment phase. Given the ethical considerations about the induction of negative mood in clinically depressed patients, like others before us, we have conducted the BFT using the first assessment phase only [40, 44]. The BFT encompassed a practice phase and the actual task. During the practice phase, participants were instructed to focus on their breathing for 20 s. Subsequently, participants were asked to maintain focus on their breath for 45 s, while acknowledging the presence of distracting intrusive thoughts. Throughout this period, a computer-generated auditory cue (beep) occurred randomly three times, with intervals ranging from 10 to 20 s. Following each auditory cue, participants were instructed to verbally articulate whether their attention was still fixated on their breath or had shifted toward an intrusive thought. In case of distraction by a thought intrusion, participants were instructed to provide a brief descriptive label (e.g., “cannot concentrate”) and categorize the nature of the thought as either negative, positive or neutral. When participants sustained focus on their breath, their response was simply the utterance of “breath” (in Dutch: “adem”). During the actual task, participants were instructed to focus their attention on their breath for a period of 5 min and responded to 12 tones at random intervals of 20–30 s in an analogous manner to the practice phase.

### 2.6. Statistical Analysis

**Figure 2:**
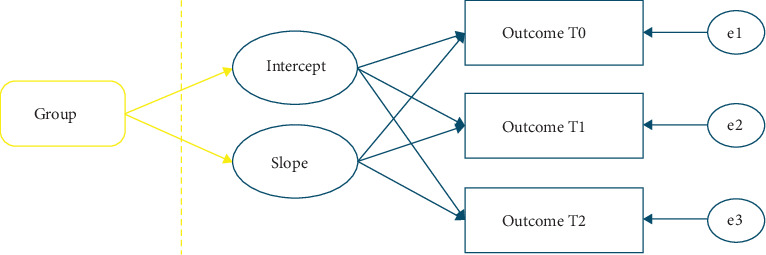
Structure of the latent growth curve models (LGCM) for the different outcome measures that were assessed before (T0), mid-way (T1) and after (T2) treatment. First unconditional models were run (blue part). Subsequently conditional models were run, with group as predictor of the latent growth factors (yellow). Group = mindfulness-based cognitive therapy + treatment as usual (TAU) versus TAU.

Statistical analyses were run according to the statistical analysis plan (NCT05802966; ClinicalTrials.gov) [45]. Independent-samples *t*-test or chi-square test were performed to compare intervention (MBCT + TAU) and control (TAU) group on demographic and baseline characteristics. The impact of treatment (MBCT + TAU versus TAU) on depressive symptoms (primary outcome), overall functional impairment (secondary outcome), and hypothesized mediators was examined within the intention-to-treat (ITT) sample using latent growth curve models (LGCM) [46]. The intercept and slope were modeled from data collected pre-MBCT/wait-list (T0), halfway MBCT/waiting period (T1) and post-MBCT/waiting period (T2) (Figure 2). Initially, unconditional models were fitted to ascertain whether a linear or nonlinear trend of change over time best suited the data. The results of those models and decsisions for LGCM model building are reported more extensively in the supporting information. In summary, the unconditional models generally showed adequate to good fit, with some models achieving improved fit after applying specific constraints (Supporting Information Table S1), which was not improved by free estimation of time scores.

In addition, to assess the rate of change within the two groups, Cohen's (*d*) within-group effect sizes were calculated by dividing the mean-difference (T2–T0) by the standard deviation (SD) of the difference, with 0.2, 0.5, and 0.8 indicating a small, medium, and large effect, respectively. Next, conditional models were run for all variables with Group as a predictor of slope and intercept, which showed good fit (Supporting Information Table S1). The path from group to intercept reflects baseline differences between groups. In addition, the path from Group to slope reflects between-group differences in the trajectories of change on the outcome measure(s). Between-group effect sizes were calculated within the context of the growth model analyses (GMA, with the following equation: $d=\frac{\beta 11\times \text{time}}{\text{Pooled SD raw scores}}$ [47], in which *β*11 is the difference between the means of the unstandardized slopes of the MBCT + TAU versus TAU group. By multiplying *β*11 with time, the difference between the model-estimated means of both groups at the end of the study (controlled for baseline differences) was obtained. This GMA (model-specific) effect size is equivalent to the Cohen's *d* calculated for independent groups with a pretest, post-test design. Potential moderators of treatment effect (self-report measures of RNT, mindfulness skills and self-compassion, and negative intrusive thoughts on the BFT) were added as predictor of the latent growth factors. A significant interaction effect among the predictor, Group, and slope would indicate moderation of treatment effect, controlled for the association between predictor and outcome at baseline. Sensitivity analyses were conducted to verify that the observed moderation effects were not confounded by the interrelations among the moderators or by borderline significant baseline characteristics, namely: country of birth, work situation, and current treatment status.

**Figure 3:**
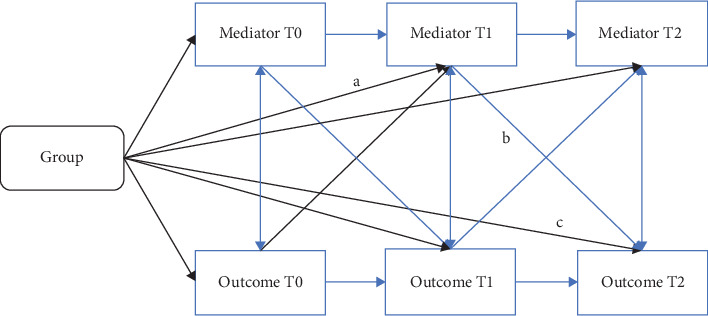
Structure of cross-lagged structural equation models (CLSEM) of mediation for outcome at post-treatment (T2) by mediator at mid-treatment (T1) (a) represents the effect of Group on mediator at mid-treatment (T1), (b) the effect of mediator at mid-treatment on outcome at post-treatment (T2), and (c) the direct path of group on outcome at post-treatment, while a*⁣*^*⁣*^*∗*^^b represents the indirect path from Group on outcome at post-treatment via mediator at mid-treatment. Group = mindfulness-based cognitive therapy (MBCT) + treatment as usual (TAU) versus TAU, T0, pretreatment; T1, mid-treatment; T2, post-treatment.

Mediation of the effect of MBCT + TAU versus TAU on depressive symptoms (IDS-SR) and overall functional impairment (OQ-45) by putative mediators of interest (measures of RNT, mindfulness skills, self-compassion) were tested by employing cross-lagged structural equation models (CLSEM; Figure 3). Because those analyses are aimed at getting a better understanding of the mechanisms of change, for which a minimal effective dose of MBCT is required, the CLSEM models were run on the per protocol sample (minimal effective dose of ≥4 sessions) [4].

Mediation of the effect of Group by putative mediators on outcome measures (IDS-SR and OQ-45) were tested by running separate CLSEM models. Those models contained the following paths: autoregression effects (stability effects) of putative mediator and outcome, within-time correlations between mediator and outcome, and longitudinal cross-lagged effects between mediator and outcome (Figure 3). In addition, it was tested whether Group (MBCT + TAU versus TAU) had a significant differential effect on mediator at T1 (path a) and on outcome at T2 (path c), and whether mediator at mid-treatment (T1) predicted outcome at post-treatment (T2; path b). Finally, to formally test mediation, the indirect effect of Group on outcome at post-treatment (T2) via mediator at mid-treatment (T1) was estimated by 5000 bootstrap samples within bootstrapping procedures [48]. Confidence intervals (95%) of indirect effects were calculated and reported. To separate within-person and between person differences, as a sensitivity analysis, CLSEM models were repeated with the inclusion of random intercepts [25, 49]. The results of these RI-CLSEM models are reported within the supporting information.

Data curation, visualization, and comparisons at baseline were performed in the open-source statistical software program R (RStudio (2019). RStudio: Integrated Development for R. RStudio, PBC, Boston, MA URL http://www.rstudio.com/). LGCM and CLSEM modeling were performed in M*plus* version 8.9 [50].

Due to the exploratory nature of the study, all original *p*-values from moderation and mediation analyses were reported without adjustments for multiple comparisons.

#### 2.6.1. Sensitivity Analyses in PROCESS

The original power calculation for this study was based on assessing mediation with the PROCESS macro [51], which indicated 174 required participants to detect mediation effects (see, ClinicalTrials.gov ID: NCT05802966) [45]. However, after start of the trial but before finalization of data collection we deliberately chose to use (and register; see NCT05802966) CLSEM as our primary analysis for mediation because CLSEM (i) is a more sophisticated method that takes into account more information about how variables of interest change over time (autoregressive and cross-lagged effects), (ii) enables modeling individual participants which allows separation of within-person processes from stable between-person differences, (iii) can handle missingness of data (anticipated to be larger because of COVID-19 pandemic) and (iv) corrects for measurement error. Similarly, we used LGCM as primary analysis to test for the effect of MBCT + TAU versus TAU on pre- to post-change in outcomes (and moderation of those effects) because LGCM is a latent variable approach that models slope and intercept from the data (pre-, mid- and posttreatment measurements), allows correction for measurement error and can handle missingness of data.

As a sensitivity analysis, moderation of the effect of MBCT on pre- to post-treatment change in outcome (baseline to post-treatment residualized change scores of IDS-SR and OQ-45) and mediation of this effect by pre- to mid-treatment residualized change in mediator variables were also assessed by means of the PROCESS macro of Hayes, Montoya, and Rockwood [51]. If any of the potential moderators reached the level of significance, PROCESS allowed us to explore Johnson and Neymans regions to assess at what value of the moderator the conditional effect of Group*⁣*^*∗*^moderator becomes significant [52]. To provide an indication of the size of the moderating effect, we split the original sample into two subsamples using the Johnson-Newman values of the moderators. Subsequently, we evaluated the between-group (MBCT + TAU versus TAU) effect sizes in these subsamples. The Cohen's *d* effect sizes were calculated by dividing the difference in the mean outcomes from pre- to post-intervention between the two groups by the pooled SD of these differences. The sensitivity moderation and mediation for which the PROCESS modeling tool was used were run in R and results (including Supporting Information Tables S3, S4, and S12) are reported within the supporting information.

## 3. Results

### 3.1. Sample Characteristics

The sample consisted of 134 patients with a diagnosis of MDD with either recurrent (with or without current depression: 60.4%) or persistent (39.6%) MDD (Table 1). Information about enrollment, allocation, adherence to MBCT, and the number of patients who completed assessments at each specific time point can be found within the consort flow chart (Figure 1). Overall, patients were mildly to severely depressed (IDS-SR Mean [SD]: 28.8 [11.5]), experienced 3 or more episodes during their lifetime, and about half had one or more comorbid disorders at the start of the study. Based on date of baseline assessment and start of MBCT, 94 out of 134 patients (70%) were assigned to the intervention group (MBCT + TAU) and 40 (30%) to the control group (TAU). At baseline, there were no significant differences in demographic and clinical characteristics, nor in any of the outcome measures (Table 1).

### 3.2. Effect of MBCT on Change in Outcomes

Scores of outcome measures in both groups at the three assessment moments (T0–T2) are presented in Table 2. At baseline, groups did not differ on all outcome measures (no significant effect of Group on intercept for all outcomes: all *p* > 0.10). Group did significantly impact change over time (slope) of both depressive symptoms, overall functional impairment, and all other assessed variables except for negative intrusive thoughts on the BFT (Tables 2 and 3). Specifically, patients in the MBCT + TAU condition showed a greater reduction in depressive symptoms (LGCM between-group ES = −0.54) and in overall functional impairment (LGCM between-group ES = −0.44) than those in the TAU condition (Tables 2 and 3). For effect sizes of other variables (range 0.31–0.58), the reader is referred to Table 2.

### 3.3. Moderation of the Effect of MBCT on Depressive Symptoms

**Figure 4:**
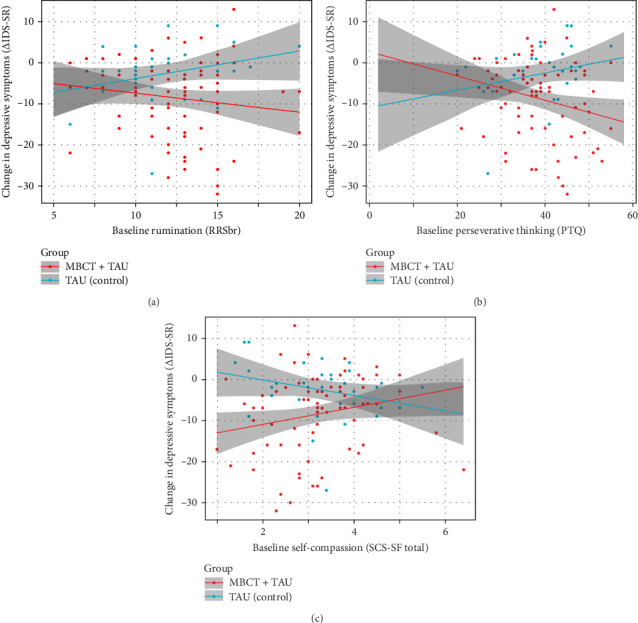
Moderation of the effect of group on rate of change in depressive symptoms by pretreatment levels of rumination, perseverative thinking and self-compassion. This figure displays change in depressive symptom severity from pre- to post-treatment in relation to pretreatment levels of (A) rumination, (B) perseverative thinking, and (C) self-compassion. Plots display scores for individual participants with a least square regression line (and 95% confidence level) for patients within the MBCT + TAU (red) and control (TAU; blue) condition. MBCT, mindfulness-based cognitive therapy, TAU, treatment us usual.

Subsequently, moderation of the effect of Group on rate of change in depressive symptoms was tested within the ITT sample for baseline levels of rumination (brooding subscale of ruminative response scale [RRSbr]), perseverative thinking (PTQ), mindfulness skills (FFMQ), self-compassion (SCS), and negative intrusive thoughts (BFT). Fit of those models for the self-report questionnaires was good. For negative intrusive thoughts, fit was acceptable (Supporting Information Table S1). Rumination, perseverative thinking, and self-compassion moderated the effect of MBCT on depressive symptoms (Table 4). This was not the case for mindfulness skills and negative intrusive thoughts (Table 4). More specifically, higher levels of rumination or perseverative thinking, or lower levels of self-compassion before treatment (T0), were associated with a greater reduction in depressive symptoms in the MBCT + TAU compared to the TAU condition (Figure 4). Moderation of the treatment effect by rumination, perseverative thinking and self-compassion were independent from each other. More specifically, moderation of the effect of Group by self-compassion remained significant when adding baseline rumination or perseverative thinking as covariates to the model (Table 4). Similarly, moderation of the effect of Group by (i) rumination and (ii) perseverative thinking remained significant when adding baseline self-compassion as covariate to the models (Table 4). Additionally, when correcting for borderline significant baseline variables (country of birth, work situation, and current treatment), moderation of the treatment effect by rumination, perseverative thinking, and self-compassion remained significant (Table 4).

### 3.4. Moderation of the Effect of MBCT on Overall Functional Impairment

The same moderation analyses were conducted with overall functional impairment (OQ-45) as outcome. In general, model fit was adequate, but root mean square error of approximation (RMSEA) values were relatively high (Supporting Information Table S1). Results were similar as those for depressive symptoms: rumination, perseverative thinking and self-compassion moderated the effect of Goup on reduction in overall functional impairment (Supporting Information Table S2/Supporting Information Figure S1). In addition, for overall functional impairment as outcome, BFT was also found to be a significant moderator (Supporting Information Table S2). Thus, higher levels of self-reported repetitive (negative) thoughts and negative thoughts on the BFT, and lower levels of self-compassion were associated with a greater reduction in overall functional impairment in MBCT + TAU compared to TAU. Similar to findings for depressive symptoms, moderation of the effect on overall functional impairment by rumination, perseverative thinking and self-compassion were independent of each other. When additionally correcting for borderline significant baseline variables (country of birth, work situation, and current treatment), results were consistent with the uncorrected results. However, moderation by rumination (*p*=0.055) and self-compassion (when also corrected for perseverative thinking: *p*=0.061) now reached *p*-values slightly above the 0.05 threshold (Supporting Information Table S2).

### 3.5. Mediation of the Effect of MBCT on Depressive Symptoms

All CLSEM models for depressive symptoms showed good to excellent fit (Supporting Information Table S5). Autoregressive effects of those CLSEM models show that depressive symptoms (IDS-SR range: 0.61–0.83) and mediator variables (range mediators: 0.59–0.81) were relatively stable over time, except for negative intrusive thoughts (range negative intrusive thoughts: 0.17–0.44), see Supporting Information Figures S2–S6. In addition, evaluation of within-time correlations of depressive symptoms (IDS-SR) and self-report mediator variables (rumination, perseveative thinking, mindfulness skills and self-compassion) reveals strong correlations at baseline (T1) and relatively weaker correlations at mid-treatment (T2) and post-treatment (T3) (for specifics see Supporting Information Figures S2–S5). Negative intrusive thoughts were less strongly related to outcomes at the different time points (Supporting Information Figure S6).

When evaluating cross-lagged effects, none of the mediator variables at mid-treatment (T1) predicted depressive symptoms (IDS-SR) at post-treatment (T2) (Table 5, Supporting Information Figures S2–S6). In addition, Group did not have an effect on mediator variables at mid-treatment (T1), and those effect sizes were all (very) small (standardized coefficients ≤0.1) In line, there was no significant indirect mediation effect of Group on depressive symptoms at post-treatment (T2) via mediating variables at mid-treatment (T1) (Table 5).

### 3.6. Mediation of the Effect of MBCT on Overall Functional Impairment

CLSEM results for the models in which overall functional impairment at T0–T2 were run as outcome were to a large extent similar as those of depressive symptoms (see Supporting Information Table S6–S8 and Supporting Information Figures S7–S11 for details). Importantly, no mediation effect of group on overall functional impairment at post-treatment (T2) via mediating variables at mid-treatment (T1) was found.

## 4. Discussion

Our objective was to identify potential moderators and mediators of MBCT in a group of patients with recurrent or persistent depression. Higher baseline levels of RNT and lower levels of self-compassion were associated with a larger reduction in depressive symptoms and overall functional impairment for the MBCT + TAU versus TAU (control) condition. Negative intrusive thoughts reported on the BFT exclusively moderated the effects of MBCT on overall functional impairment. In contrast to our hypothesis, there was no significant effect of MBCT on assessed mediators at mid-treatment, and mediators at mid-treatment did not predict post-treatment outcomes. In line with those findings, pretreatment to mid-treatment change in putative mediator variables did not mediate the effect of MBCT on depressive symptoms and overall functional impairment at post-treatment.

### 4.1. Moderation

This moderation effect of rumination on the effect of MBCT on depressive symptoms in patients with recurrent and persistent depression is in line with two previous reports. Cladder-Micus et al. [19] also found that pretreatment levels of rumination moderated the effect of MBCT + TAU versus TAU on depressive symptoms in persistent treatment-resistant depression. In addition, a tree-based qualitative interaction analysis of individual patient data (IPD) from three RCTs in recurrent or persistent MDD showed that MDD patients with an earlier onset and higher pretreatment rumination benefited more from MBCT + TAU [20]. Our study extended prior research by demonstrating that moderation applies to both a content-dependent measure (RRS brooding subscale: [35]) and a content-independent measure of RNT (PTQ: [36]), highlighting the importance of both depression-related content and the perseverative cognitive process itself. Finding the same moderation effects for depressive symptoms and overall functional impairment as an outcome strengthens the robustness of those results.

A further advancement from the current study is the inclusion of a task-based state measure of RNT. The majority of studies that investigated the workings of MBCT relied on self-report measures, which entail risk of recall bias and common variance bias [53]. Kazdin [23] already called for triangulation of self-report, experimental, and neuroscienctific evidence for a better understanding of the mechanisms involved in psychological treatments. The self-report and experimental measures of RNT in our study pointed in the same direction. Although baseline levels of experimentally measured negative intrusive thoughts did not moderate the effect of MBCT on depressive symptoms (*p*=0.1), they did moderate the effect on overall functional impairment as measured with the OQ-45. Subsequent research should replicate these findings to validate the robustness of this measure and assess its practical utility.

In addition to RNT, we found lower levels of self-compassion before treatment to be a significant moderator of the effects of MBCT as well. This might implicate that MBCT is especially beneficial for patients showing a harsh and self-critical, judgmental attitude when facing failures or difficulties. Note that a previous comparable study in persistent, treatment-resistant depression [19] reported no significant moderating effect of self-compassion. Nevertheless, the ITT sample in which they assessed moderation was smaller (*n* = 89) and they did find a trend toward significance; *p* = 0.067). Whether their findings are different from our findings is therefore an open question. Further research, particularly studies with larger samples that allow for more robust subgroup analyses, might further our insights here. These studies might further help to clarify whether the moderating effect of self-compassion varies by the type and chronicity of depressive disorders. In addition, future research is needed to determine whether perseverative thinking, rumination and self-compassion could also reflect possible overlapping or proxy variables such as levels of engagement or readiness for change [54]. This will enhance our understanding of how these factors interact and influence MBCT-induced reduction in depressive symptoms.

Future studies could experimentally test whether allocation based on pretreatment rumination and self-compassion would indeed increase effectiveness of MBCT. The Johnson–Neyman regions of significance reported in the current manuscript might be indicative for thresholds. These revealed that patients who score 9 points or higher on the RRS brooding subscale, or 4 points or lower on the SCS-SF, will be more likely to benefit from MBCT in terms of depressive symptom reduction. Moreover, the between-group effect size (MBCT + TAU versus TAU) for depressive symptom reduction was moderate to large in patients with a rumination score of 9 or higher. In contrast, it was small, in the opposite direction and nonsignificant for patients with a rumination score below 9. Similarly, the effect size for patients with a self-compassion score of 4 or lower was moderate to large, while it was small and nonsignificant for those with a self-compassion score above 4. These effect sizes indicate that the differential treatment effect was substantial for a significant portion of the sample, and suggests that Johnson-Newman values could be useful for selecting patients for MBCT. Although first steps have been made in identifying potential moderators of MBCT for depression that may assist in effective treatment allocation, caution should be exercised to preselect patients solely based on these results.

Furthermore, identification of treatment moderators may also help in identifying mediators of treatment effect [23]. For example, investigating mediators of the effects of MBCT (versus control) within a more homogeneous group of only high-ruminators may help in uncovering mediation-effects. Future studies into the workings of MBCT for depression may benefit from solely including patients with a cutoff score on rumination and self-compassion, which may increase the likelihood of establishing mediation effects.

### 4.2. Mediation

Almost all previous studies that investigated mediation assessed potential mediators and outcomes at pre- and post-treatment only, which prevented conclusions about the temporal order of change [10, 11]. We advanced previous research by including measurements half-way the MBCT/control period and assessing mediation by means of (RI) CLSEM panel models. This allowed assessing the interplay between (change in) mediator and outcome in a longitudinal context and formally testing indirect paths from Group to outcome at post-treatment via potential mediators at mid-treatment.

It seems unlikely that our null-findings with respect to mediation were caused by a lack of statistical power, especially because mid-treatment effects of Group on mediators are far from significant with very small coefficient *β* effect sizes (≤0.10). Moreover, our study had enough statistical power to detect medium-sized effects for mediation analysis within a panel model context [55].

A similar CLSEM approach with pre-, mid-, and post-treatment measurements was performed by Spinhoven et al. [56] in a study of MBCT for treatment-refractory anxiety disorder. Of all putative mediators (i.e., mindfulness, worry, rumination, and difficulties in emotion regulation), MBCT (versus Cognitive Behavioral Therapy) only affected emotion regulation at mid-treatment and no significant indirect mediation paths were found [56]. In addition, within the context of an RCT of MBCT (versus active control) for persistent treatment-resistant depression, Eisendrath et al. [24] did not find differences between conditions in the putative mediators assessed at mid-treatment (i.e., mindfulness, self-compassion, rumination and experiential avoidance), and no mediation effect was established. Moreover, other studies that measured at mid-treatment point in the same direction: no difference between MBI and control conditions in potential mediators at mid-treatment [57, 58]. Thus, measuring at mid-treatment may be too early for meaningful mediation analyses because no difference between control and MBCT group has yet arisen (no significant a-path, with small effect sizes).

### 4.3. Limitations and Directions for Future Research

This study also has several important limitations. First, patients were not randomly assigned to condition; they were allocated based on the time between the baseline clinical assessment and the start date of MBCT. This resulted in unbalanced inclusion in the two arms of this trial, with more patients allocated to the intervention arm (MBCT + TAU), which may have influenced the statistical power. Indeed, the variance of a dichotomous categorical moderator variable (e.g., Group) is maximized when groups are equal [59]. In the case of unequal groups, moderation effects may be underestimated [59]. But, even in those potentially imperfect conditions, we found interaction effects between Group and RNT, and Group and self-compassion. Another potential disadvantage of assignment based on clinical assessment date is unintentional introduction of differences between the intervention and control group. However, at the beginning of the study, there were no significant differences between the two groups. Another limitation of this study is the lack of systematic monitoring of what regular clinical practice (TAU) entailed throughout the study period. Future studies would benefit from consistently tracking TAU to better control for variations in TAU and to provide a more accurate assessment of both treatment and moderation effects. In addition, the study's follow-up period was limited to the duration of the intervention, and therefore, (moderation of) potential medium-to-long term effects could not be evaluated. However, other studies in depression with a longer follow-up showed that MBCT-induced depressive symptom reduction remained stable after 6-months [60] and 1 year follow-up [61], but see [5]. Lastly, while inclusion of the BFT to triangulate methods of assessment is a strength [62], the BFT also has an important limitation. Zero-inflated outcomes observed on the BFT (as discussed in [44]) may complicate finding associations between baseline RNT and treatment effect (moderation) and may restrict interpretation of BFT outcomes. In addition, although MBCT was delivered by certified teachers who met the advanced criteria of the Association of Mindfulness-Based Teachers in the Netherlands and Flanders (which are in concordance with the Good Practice guidelines of the UK Network of Mindfulness-Based Teacher Trainers), and MBCT was supervised by a highly experienced teacher—a notable strength of the study—we acknowledge the lack of formal assessments of teacher competence and adherence to the MBCT manual as a limitation. Future research comparing MBCT with another evidence-based treatment such as CBT could investigate whether these moderation effects persist across different therapeutic approaches. In addition, future research may benefit from assessing at what moment in time (e.g., weekly measures at each MBCT session/week) change in mediators and outcome first appear. Additionally, examining mediation in a more intensive longitudinal context may improve our understanding of mechanisms of change on a smaller time scale that would be unattainable with less frequent data collection [63]. Studies employing these methods are emerging and exemplify indeed that establishing the moment of change in putative mediator (and outcome) variables [64] and assessing mediation in a more longitudinal context (e.g., weekly measures; [65, 66], or ecological momentary assessment; [67, 68]) may help in uncovering the workings of MBIs.

Furthermore, there is accumulating evidence that RNT is a transdiagnostic process and that higher levels of RNT are present across many psychiatric disorders, such as mood and anxiety disorders [69–71]. In addition, Spinhoven et al. [70, 71] showed that common rather than unique aspects of RNT are related to depression and anxiety disorders as well as persistence and relapse of those disorders. So because of this transdiagnostic role of RNT, and its importance in various disorders, it may be worth investigating whether higher levels of RNT also moderate MBCT-induced effects in patients with anxiety disorder and other psychiatric disorders.

### 4.4. Conclusions

Our results indicate that MBCT might be especially beneficial for patients with recurrent or persistent MDD who ruminate a lot and exhibit lower levels of self-compassion. Confirmation of those moderators in future research, preferably by IPD meta-analyses, is warranted. Identification of these moderators may inform (shared) decision making on treatment allocation for MBCT.

Despite the methodological improvements of this study, no mediating effects of rumination, perseverative thinking, mindfulness skills, or self-compassion were found. Nevertheless, an advanced understanding of the mechanisms of MBCT could be helpful for further optimizing treatment efficacy. Large, well-designed studies with longitudinal designs characterized by more frequent data collection and enriched with qualitative interviews and additional neurocognitive assessments might provide a more fruitful approach to uncover mechanisms at play in MBCT.

## Figures and Tables

**Table 1:** Demographic and clinical characteristics and outcome measures at baseline compared between intervention (MBCT + TAU) and control (TAU) group.

	MBCT + TAU (*n* = 94)	TAU (control) (*n* = 40)	Total (*n* = 134)	*p* Value
Age	—	—	—	0.306^a^
Mean (SD)	44.9 (14.1)	47.5 (12.5)	45.6 (13.6)	—
Gender–female (%)	56 (59.6%)	24 (60.0%)	80 (59.7%)	0.963^b^
Country of birth—Netherlands (%)	86 (91.5%)	32 (80.0%)	118 (88.1%)	0.061^b^
Marital status	—	—	—	0.971^c^
Married/registered partnership	31 (33.0%)	14 (35.0%)	45 (33.6%)	—
Living together	19 (20.2%)	8 (20.0%)	27 (20.1%)	—
Unmarried/never been married	33 (35.1%)	13 (32.5%)	46 (34.3%)	—
Divorced	9 (9.6%)	5 (12.5%)	14 (10.4%)	—
Widow/widower	2 (2.1%)	0 (0.0%)	2 (1.5%)	—
Education	—	—	—	0.394^c^
Lower	4 (4.3%)	0 (0.0%)	4 (3.0%)	—
Intermediate	21 (22.3%)	7 (17.5%)	28 (20.9%)	—
Higher	69 (73.4%)	33 (82.5%)	102 (76.1%)	—
Work situation	—	—	—	0.071^c^
Employed/student/homemaker	61 (65.6%)^1^	33 (82.5%)	94 (70.7%)^1^	—
(Early) retirement	4 (4.3%)	2 (5.0%)	6 (4.5%)	—
Unemployed	28 (30.1%)	5 (12.5%)	33 (24.8%)	—
Depressive symptoms (IDS-SR)	—	—	—	0.998^a^
Mean (SD)	28.8 (12.8)^1^	28.8 (8.0)	28.8 (11.5)^1^	—
Overall functional impairment (OQ-45)	—	—	—	0.688^a^
Mean (SD)	74.7 (20.7)^1^	76.2 (17.1)	75.2 (19.6)^1^	—
RRSbr	—	—	—	0.071^a^
Mean (SD)	12.6 (3.2)^1^	11.5 (2.8)	12.2 (3.1)^1^	—
PTQ total	—	—	—	0.423^a^
Mean (SD)	37.5 (9.4)^1^	38.9 (7.6)	38.0 (8.9)^1^	—
FFMQ-SF total	—	—	—	0.262^a^
Mean (SD)	14.8 (2.7)^1^	15.4 (2.1)	15.0 (2.6)^1^	—
SCS-SF total	—	—	—	0.574^a^
Mean (SD)	3.2 (1.0)^1^	3.3 (1.0)	3.3 (1.0)^1^	—
STAI	—	—	—	0.624^a^
Mean (SD)	55.1 (9.7)^1^	54.3 (7.0)	54.9 (8.9)^1^	—
Negative intrusive thoughts (BFT)	—	—	—	0.266^d^
Median	1.5^16^	2.0^7^	2.0^23^	—
Q1, Q3	0.0, 3.0	0.0, 4.0	0.0, 3.5	—
Depression type and status	—	—	—	0.825^c^
Recurrent remission (partial)	18 (19.1%)	10 (25.0%)	28 (20.9%)	—
Recurrent remission (full)	15 (16.0%)	5 (12.5%)	20 (14.9%)	—
Recurrent remission (unknown type)	2 (2.1%)	1 (2.5%)	3 (2.2%)	—
Persistent depressive disorder	36 (38.3%)	17 (42.5%)	53 (39.6%)	—
Recurrent current	23 (24.5%)	7 (17.5%)	30 (22.4%)	—
Age start current depression	—	—	—	0.648^a^
Mean (SD)	44.0 (14.1)	45.8 (15.6)	44.5 (14.5)	—
Duration current depression (months)	—	—	—	0.964^d^
Median	11.5	9.0	10.0	—
Q1, Q3	4.8, 26.2	2.5, 31.0	3.5, 28.5	—
Duration current remission (months)	—	—	—	0.744^d^
Median	5.0	4.0	5.0	—
Q1, Q3	2.0, 9.0	2.0, 11.0	2.0, 9.5	—
Duration last episode (if cur rem) (months)	—	—	—	0.550^d^
Median	6.0	7.5	6.5	—
Q1, Q3	2.6, 11.5	3.5, 17.2	2.6, 12.0	—
Episodes lifetime	—	—	—	0.303^d^
Median	3.0^5^	3.0^3^	3.0^8^	—
Q1, Q3	2.0, 5.0	2.0, 4.0	2.0, 5.0	—
Age first depression	—	—	—	0.968^a^
Mean (SD)	25.1 (12.1)^1^	25.0 (14.1)^4^	25.1 (12.7)^5^	—
Number of comorbid disorders	—	—	—	0.421^c^
Three or more comorbid disorders	3 (3.2%)	4 (10.0%)	7 (5.2%)	—
Two comorbid disorders	10 (10.6%)	5 (12.5%)	15 (11.2%)	—
One comorbid disorder	35 (37.2%)	12 (30.0%)	47 (35.1%)	—
No comorbid disorder	46 (48.9%)	19 (47.5%)	65 (48.5%)	—
Current treatment	—	—	—	0.067^c^
Medication and psychotherapy	31 (33.0%)	15 (37.5%)	46 (34.3%)	—
Medication	31 (33.0%)	6 (15.0%)	37 (27.6%)	—
Psychotherapy	15 (16.0%)	5 (12.5%)	20 (14.9%)	—
No medication or psychotherapy	17 (18.1%)	14 (35.0%)	31 (23.1%)	—
MBCT attended (0–9)	—	—	—	0.912^a^
Mean (SD)	7.6 (1.9)	7.6 (2.3)^1^	7.6 (2.0)^1^	—

Abbreviations: FFMQ-SF, five facet mindfulness questionnaire-short-form; IDS-SR, inventory of depressive symptomatology-self-report; MBCT, mindfulness-based cognitive therapy; OQ-45, outcome questionnaire-45; PTQ, perseverative thinking questionnaire; RRSbr, brooding subscale of ruminative response scale; SCS-SF, self compassion scale-short-form; STAI, state-trait anxiety inventory; TAU, treatment as usual.

^a^Linear model ANOVA, ^b^Pearson's Chi-squared test, ^c^Fisher's Exact test, ^d^Kruskal–Wallis rank sum test; ^1^1 missing, ^3^3 missing, ^4^4 missing, ^5^5 missing, ^7^7 missing ^8^8 missing, ^16^16 missing, ^23^23 missing.

**Table 2:** Outcome measures at different time points and within- and between-group effect sizes.

Outcomes	Time points	MBCT + TAU	TAU (control)	Pre- to posttreatment difference (T0-T2) in mean-change	LGCM between group effect-size (T0–T2)
		Mean (SD)		Mean-change (pooled SD)	—
Depressive symptoms (IDS-SR)		—		—	—
	T0	28.8 (12.8)	28.8 (8.0)	—	—
	T1	23.6 (11.5)	27.1 (10.9)	—	—
	T2	19.8 (11.3)	27.1 (10.6)	−5.8 (11.5)	*−0.54*
*Within group cohens d* (*T0-T2*)		*0.89*	*0.39*	—	—
	T3	—	25.2 (10.9)	—	—
	T4	—	21.1 (9.2)	—	—
*Within group cohens d* (*T2-T4*)		—	*0.81*	—	—
		—	—	—	—
Overall functional impairment (OQ-45)		—	—	—	—
	T0	75.7 (20.7)	76.2 (17.1)	—	—
	T1	70.8 (62.7)	77.1 (23.0)	—	—
	T2	62.7 (21.9)	74.1 (22.8)	−8.6 (19.7)	*−0.44*
*Within group cohens d* (*T0-T2*)		*0.74*	*0.24*	—	—
	T3	—	71.6 (22.0)	—	—
	T4	—	63.7 (21.3)	—	—
*Within group cohens d* (*T2-T4*)		—	*0.83*	—	—
		—	—	—	—
Brooding (RRS brooding subscale)		—	—	—	—
	T0	12.6 (3.2)	11.5 (2.8)	—	—
	T1	11.0 (2.8)	11.2 (3.5)	—	—
	T2	10.2 (3.2)	10.9 (3.1)	−1.2 (3.1)	*−0.43*
*Within group cohens d* (*T0-T2*)		*0.74*	*0.43*	—	—
	T3	—	10.6 (2.9)	—	—
	T4	—	10.1 (2.8)	—	—
*Within group cohens d* (*T2-T4*)		—	*0.51*	—	—
	—	—	—	—	—
Perseverative thinking (PTQ)		—	—	—	—
	T0	37.5 (9.4)	38.9 (7.6)	—	—
	T1	35.2 (10.4)	36.8 (9.3)	—	—
	T2	31.3 (10.0)	35.9 (10.2)	−3.2 (8.9)	*−0.31*
*Within group cohens d* (*T0-T2*)		*0.73*	*0.52*	—	—
	T3	—	34.0 (10.7)	—	—
	T4	—	30.6 (10.7)	—	—
*Within group cohens d* (*T2-T4*)		—	*0.80*	—	—
		—	—	—	—
Mindfulness skills (FFMQ-SF)		—	—	—	—
	T0	14.8 (2.7)	15.4 (2.1)	—	—
	T1	15.5 (2.5)	15.6 (2.4)	—	—
	T2	16.5 (2.4)	15.9 (2.6)	1.2 (2.6)	*0.48*
*Within group cohens d* (*T0-T2*)		*0.72*	*0.25*	—	—
	T3	—	15.9 (2.4)	—	—
	T4	—	17.2 (2.5)	—	—
*Within group cohens d* (*T2-T4*)		—	*1.07*	—	—
		—	—	—	—
Self-compassion (SCS-SF)		—	—	—	—
	T0	3.2 (1.0)	3.3 (1.0)	—	—
	T1	3.4 (1.0)	3.4 (1.1)	—	—
	T2	3.8 (1.1)	3.5 (1.1)	0.44 (1.0)	*0.43*
*Within group cohens d* (*T0-T2*)		*0.71*	*0.21*	—	—
	T3	—	3.7 (1.2)	—	—
	T4	—	4.0 (1.2)	—	—
*Within group cohens d* (*T2-T4*)		—	*0.74*	—	—
		—	—	—	—
Anxiety (STAI)		—	—	—	—
	T0	55.1 (9.7)	54.3 (7.0)	—	—
	T1	51.4 (10.8)	53.0 (9.0)	—	—
	T2	48.2 (10.1)	53.2 (9.6)	−5.1 (9.0)	*−0.58*
*Within group cohens d* (*T0-T2*)		*0.82*	*0.20*	—	—
	T3	—	51.6 (8.8)	—	—
	T4	—	48.7 (8.3)	—	—
*Within group cohens d* (*T2-T4*)		—	*0.73*	—	—
		—	—	—	—
Negative intrusive thoughts (BFT)		—	—	—	—
	T0	2.10 (2.30)	2.73 (2.59)	—	—
	T1	1.63 (2.11)	1.93 (2.62)	—	—
	T2	1.11 (1.40)	1.22 (1.78)	—	0.21 (ns)
*Within group cohens d* (*T0-T2*)		*0.52*	*0.75*	—	—
	T3	—	0.90 (1.30)	—	—
	T4	—	0.77 (0.81)	—	—
*Within group cohens d* (*T2-T4*)		—	*0.32*	—	—

*Note:* Means and standard deviations of outcome measures are presented for all participants who completed the measurement at a specific time point.

Abbreviations: BFT, negative intrusive thoughts reported on the breathing focus task; FFMQ-SF, five facet mindfulness questionnaire-short-form; IDS-SR, inventory of depressive symptomatology-self-report; MBCT, mindfulness-based cognitive therapy; OQ-45, outcome questionnaire-45; PTQ, perseverative thinking questionnaire; RRSbr, brooding subscale of ruminative response scale; SCS-SF, self compassion scale-short-form; STAI, state–trait anxiety inventory; TAU, treatment as usual.

**Table 3:** Model parameter estimates for rate of change (slope) in unconditional models and for group as predictor of rate of change in conditional models.

Outcomes	*Mean of slope* mean [95 CI]	*p*	*Slope on group* mean [95 CI]	*p*
IDS-SR	−3.32 [−4.14, −2.51]	<0.001	−3.14 [−4.62, −1.66]	<0.001
OQ−45	−4.86 [−6.36, −3.37]	<0.001	−4.37 [−6.89, −1.86]	0.001
RRSbr	−0.86 [−1.10, −0.62]	<0.001	−0.67 [−1.16, −0.19]	0.006
PTQ	−2.69 [−4.42, −1.97]	<0.001	−1.40 [−2.78, −0.02]	0.047
FFMQ-SF	0.70 [0.49, 0.91]	<0.001	0.62 [0.21, 1.02]	0.003
SCS-SF	0.24 [0.17, 0.32]	<0.001	0.22 [0.08, 0.37]	0.002
STAI	−2.59 [−3.30, −1.87]	<0.001	−2.62 [−3.91, −1.33]	<0.001
BFT	−0.56 [−0.81, −0.31]	<0.001	0.25 [−0.34, 0.85]	0.41

Abbreviations: BFT, negative intrusive thoughts reported on the breathing focus task; FFMQ-SF, five facet mindfulness questionnaire-short-form; Group, mindfulness-based cognitive therapy (MBCT) + treatment as usual (TAU) versus TAU; IDS-SR, inventory of depressive symptomatology-self-report; OQ-45, outcome questionnaire-45; PTQ, perseverative thinking questionnaire; RRSbr, brooding subscale of ruminative response scale; SCS-SF, self compassion scale-short-form; STAI, state–trait anxiety inventory.

**Table 4:** Parameter estimates of conditional latent growth models of depressive symptoms moderated by measures of RNT, mindfulness skills, and self-compassion.

Moderator	Unstandardized coefficient [95 CI]	*p*-Value
*RRS brooding subscale as moderator*		
Slope on group	3.24 [−1.71, 8,20]	0.20
Slope on RRSbr	0.73 [0.08, 1.39]	0.027
Slope on group*⁣*^*∗*^RRSbr	−0.55 [−0.94, −0.11]	0.014
*Slope on group*⁣*^*∗*^RRSbr corrected for SCS-SF*	*−0.52* [*−0.94*, *−0.10*]	*0.015*
*Slope on group*⁣*^*∗*^RRSbr corrected for SCS-SF*, *Ccountry of birth*, *work situation and current treatment*	*−0.50* [*−0.91*, *−0.10*]	*0.014*
*PTQ as moderator*		
Slope on group	6.10 [−1.09, 13.3]	0.10
Slope on PTQ	0.33 [0.02, 0.64]	0.037
Slope on group*⁣*^*∗*^PTQ	−0.24 [−0.42, −0.06]	0.008
*Slope on group*⁣*^*∗*^PTQ corrected for SCS-SF*	*−0.25* [*−0.43*, *−0.07*]	*0.008*
*Slope on group*⁣*^*∗*^PTQ corrected for SCS-SF*, *country of birth*, *work situation and current treatment*	*−0.23* [*−0.41*, *−0.04*]	*0.016*
*FFMQ-SF as moderator*	—	—
Slope on group	−9.57 [−20.1, 0.96]	0.075
Slope on FFMQ-SF	−0.52 [−1.63, 0.60]	0.36
Slope on group*⁣*^*∗*^FFMQ-SF	0.42 [−0.24, 1.08]	0.21
*Slope on group*⁣*^*∗*^FFMQ-SF corrected for SCS-SF*, *country of birth*, *work situation and current treatment*	*0.35* [*−0.32*, *1.02*]	*0.30*
*SCS-SF as moderator*		
Slope on group	−9.37 [−14.3, −4.45]	< 0.001
Slope on SCS-SF	−2.62 [−4.71, −0.52]	0.014
Slope on group*⁣*^*∗*^SCS-SF	1.92 [0.47, 3.36]	0.009
*Slope on group*⁣*^*∗*^SCS-SF corrected for RRSbr*	*1.94* [*0.51*, *3.37*]	*0.008*
*Slope on group*⁣*^*∗*^SCS-SF corrected for RRSbr*, *country of birth*, *work situation and current treatment*	*1.78* [*0.23*, *3.32*]	*0.024*
*Slope on group*⁣*^*∗*^SCS-SF corrected for PTQ*	*1.88* [*0.48*, *3.29*]	*0.009*
*Slope on group*⁣*^*∗*^SCS-SF corrected for PTQ*, *country of birth*, *work situation and current treatment*	*1.70* [*0.17*, *3.22*]	*0.030*
*Negative intrusive thoughts* (*BFT*) *as moderator*		
Slope on group	−1.88 [−4.01, 0.26]	0.085
Slope on BFT	0.68 [−0.40, 1.75]	0.22
Slope on group*⁣*^*∗*^BFT	−0.60 [−1.31, 0.12]	0.10
*Slope on group*⁣*^*∗*^BFT corrected for SCS-SF*, *country of birth*, *work situation and current treatment*	*−0.62* [*−1.32*, *0.09*]	*0.09*

Abbreviations: BFT, negative intrusive thoughts reported on the breathing focus task; FFMQ-SF, five facet mindfulness questionnaire-short-form; Group, mindfulness-based cognitive therapy (MBCT) + treatment as usual (TAU) versus TAU; IDS-SR, inventory of depressive symptomatology-self-report; OQ-45, outcome questionnaire-45; PTQ, perseverative thinking questionnaire; RRSbr, brooding subscale of ruminative response scale; SCS-SF, self compassion scale-short-form; STAI, state–trait anxiety inventory.

**Table 5:** Cross-lagged structural equation model output for the mediation pathways with depressive symptoms (IDS-SR) at post-treatment (T2) as outcome.

Mediator	Predictor	Outcome	Path	*β*	95% CI	*p*
RRSbr	Group	RRSbr T1	a	−0.10	[−0.24, 0.03]	0.14
	RRSbr T1	IDS-SR T2	b	0.009	[−0.12, 0.14]	0.89
	Group	IDS-SR T2	c	−0.19	[−0.29, −0.09]	<0.001
	Group via RRSbr T1	IDS-SR T2	ab	−0.00	[−0.01, 0.01]	0.89

PTQ	Group	PTQ T1	a	0.03	[−0.07, 0.14]	0.55
	PTQ T1	IDS-SR T2	b	−0.05	[−0.22, 0.12]	0.56
	Group	IDS-SR T2	c	−0.19	[−0.28, −0.09]	<0.001
	Group via PTQ T1	IDS-SR T2	ab	−0.002	[−0.10, 0.01]	0.69

FFMQ	Group	FFMQ T1	a	0.05	[−0.08, 0.18]	0.44
	FFMQ T1	IDS-SR T2	b	−0.02	[−0.18, 0.14]	0.84
	Group	IDS-SR T2	c	−0.19	[−0.29, −0.09]	<0.001
	Group via FFMQ T1	IDS-SR T2	ab	−0.00	[−0.01, 0.01]	0.84

SCS	Group	SCS T1	a	0.05	[−0.06, 0.16]	0.37
	SCS T1	IDS-SR T2	b	−0.01	[−0.14, 0.13]	0.92
	Group	IDS-SR T2	c	−0.19	[−0.29, −0.08]	<0.001
	Group via SCS T1	IDS-SR T2	ab	0.00	[−0.01, 0.01]	0.92

BFT	Group	BFT T1	a	0.05	[−0.16, 0.26]	0.66
	BFT T1	IDS-SR T2	b	−0.04	[−0.13, 0.06]	0.43
	Group	IDS-SR T2	c	−0.19	[−0.29, −0.09]	<0.001
	Group via BFT T1	IDS-SR T2	ab	−0.00	[−0.01, 0.01]	0.67

Abbreviations: *β*, standardized coefficient; BFT, negative intrusive thoughts reported on the breathing focus task; FFMQ-SF, five facet mindfulness questionnaire-short-form; Group, mindfulness-based cognitive therapy (MBCT) + treatment as usual (TAU) versus TAU; IDS-SR, inventory of depressive symptomatology-self-report, OQ-45, outcome questionnaire 45; PTQ, perseverative thinking questionnaire; RRSbr, brooding subscale of ruminative response scale; SCS-SF, self compassion scale-short-form; STAI, state–trait anxiety inventory.

## Data Availability

The data and M*plus* code for the performed analysis are openly available in the Radboud Data Repository at (https://doi.org/10.34973/a8db-kv36).

## Supporting Information

Supporting Information Considerations, and decisions related to latent growth curve model (LGCM) construction and model fit assessment, along with the corresponding tables and figures, are presented in the “Supporting Information_DA_final” file. Additionally, sensitivity analyses for moderation and mediation analyses, along with their corresponding supporting information tables and figures, are reported in the Supporting Information as well. First, sensitivity analyses for moderation within the PROCESS macro of Hayes et al. [51] are reported. Second, sensitivity analyses for mediation analyses are reported: results of (i) Random-Intercept CLSEM models and (ii) mediation analyses using PROCESS [51] are reported. To improve the readability and maintain focus in the main manuscript, we decided to include tables and figures related to the secondary outcome (overall functional impairment) within the Supporting Information. For the same reason, all figures that display the parameter estimates of the CLSEM panel models are presented within the Supporting Information as well.
