# Supplementary material for: Rumination and Self-Compassion Moderate Mindfulness-Based Cognitive Therapy for Patients With Recurrent and Persistent Major Depressive Disorder: A Controlled Trial
Source: Depress Anxiety. 2024 Nov 25;2024:3511703. doi: 10.1155/da/3511703 (PMC11918904; doi:10.1155/da/3511703)
Supplement: Supporting Information — Considerations, and decisions related to latent growth curve model (LGCM) construction and model fit assessment, along with the corresponding tables and figures, are presented in the “Supporting Information_DA_final” file. Additionally, sensitivity analyses for moderation and mediation analyses, along with their corresponding supporting information tables and figures, are reported in the Supporting Information as well. First, sensitivity analyses for moderation within the PROCESS macro of Hayes et al. [51] are reported. Second, sensitivity analyses for mediation analyses are reported: results of (i) Random-Intercept CLSEM models and (ii) mediation analyses using PROCESS [51] are reported. To improve the readability and maintain focus in the main manuscript, we decided to include tables and figures related to the secondary outcome (overall functional impairment) within the Supporting Information. For the same reason, all figures that display the parameter estimates of the CLSEM panel models are presented within the Supporting Information as well. [file 3511703.f1.docx]

# Rumination and Self-Compassion Moderate Mindfulness-Based Cognitive Therapy for Patients with Recurrent and Persistent Major Depressive Disorder: A Controlled-Trial

# Supplementary Materials

# Results

## LGCM model building and assessment of model fit

The linear trend for the unconditional model for depressive symptoms showed good fit (Supplementary Table S1). The unconditional model for overall functional impairment showed acceptable fit, which improved when Group was added as predictor of Slope and Intercept.

For all linear growth models of overall functional impairment (OQ-45) and anxiety (STAI), the variances of the variable at T1 and T2 were restricted to be equal because of a negative residual variance at T2 upon free estimation of variances. For models with FFMQ as outcome, the variance at T2 had to be put to zero for successful convergence and good model fit. Similarly, for the model with perseverative thinking (PTQ) as outcome, the variance of T0 was put to zero for successful model convergence. After successful convergence, models showed adequate to good fit for all outcomes (Supplementary Table S1). Model fit was not improved by free estimation of time scores.

In a latent growth curve model (LGCM), the residual variances represent the amount of variance in the observed variables that is not accounted for by the latent growth factors. When the residual variances are constrained to be equal across time points, it means that the amount of unexplained variance is assumed to be the same at each time point. This assumption can be useful when there is no theoretical reason to expect that the amount of unexplained variance would differ across time points. If there is a group (intervention) variable that predicts slope and intercept, it might make sense to restrict the residual variances of the observed variables (T0-T2) to be invariant if you have reason to believe that the intervention variable affects all three time points in a similar way. However, if you expect that the intervention variable has different effects at different time points, then it does not make sense to constrain the residual variances to be equal across time points. Thus, we may restrict time point 1 and 2 to be equal (which we did if necessary for model convergence), and estimate 0 freely.

In some instances, to achieve successful model convergence in our LGCMs, we fixed the residual variance at T0 or T2 to zero (as described above). This assumes that the measurement of variable at T0/T2 is completely error-free, which is unlikely to be true in practice. Although this approach is not ideal, it was employed solely as a necessary measure to facilitate model convergence. Sensitivity moderation analyses using PROCESS

Results of the sensitivity analyses using the PROCESS macro of Hayes et al. (2017) are consistent with the LGCM results. The only exception was that rumination as moderator of treatment effect on residualized change in depressive symptoms and overall functional impairment did not reach significance anymore (IDS-SR: *p* = .078; OQ45: *p* = .14*),* for details, see Supplementary Table S3-S4). Exploration of Johnson-Neyman regions revealed that from a rumination (RRSbr) score of ≥ 8.7, a perseverative thinking (PTQ) score of ≥ 33.7 and a self-compassion (SCS-SF) score of ≤ 4 the Group*moderator interactions became significant for residualized change in depressive symptoms as outcome. Next, for ease of interpretation of this result, we evaluated between-group (MBCT + TAU versus TAU) Cohen’s d effect sizes in two subsamples generated by splitting the original sample using the Johnson-Newman values of the moderators. When the sample was split by the Johnson-Newman value of the RRSbr subscale (RRSbr ≥ 8.7: n = 118; RRSbr < 8.7: n = 15), Cohen’s d within the subsample with higher levels of rumination was moderate to large (d [95% CI] = -0.76 [-1.2, -0.32]), while it was small, in the opposite direction and non-significant in the subsample with lower levels of rumination (d [95% CI] = 0.12 [-1.0, 1.3]). Similarly, when the sample was split based on the Johnson-Newman value of the PTQ (PTQ ≥ 33.7: n = 98; PTQ < 33.7: n = 35), Cohen’s d within the subsample with higher levels of perseverative thinking was large (d [95% CI] = -0.83 [-1.3, -0.34]), while it was small and non-significant in the subsample with lower levels of perseverative thinking (d [95% CI] = -0.15 [-0.96, 0.66]). In the case of a sample split based on the Johnson-Newman value of the SCS (SCS-SF ≤ 4.0: n = 109; SCS-SF > 4.0: n = 24), Cohen’s d within the subsample with lower levels of self-compassion was moderate to large (d [95% CI] = -0.77 [-1.2, -0.31]), while it was small and non-significant in the subsample with higher levels of self-compassion (d [95% CI] = -0.15 [-1.1, 0.80]).

## Sensitivity analyses of mediation: Random-Intercept CLSEM and PROCESS

When within-person processes were separated from stable between-person differences by inclusion of random intercepts, the RI-CLSEM models with FFMQ as mediator did not converge because of a negative (non-significant) variance for the Random Intercept FFMQ between-component. This indicates that the FFMQ variable is not characterized by stable between-person differences. In other words, patients vary around the same mean or trend. As suggested by Hamaker (Mplus Discussion Forum, 2018: <http://www.statmodel.com/discussion/messages/11/25297.html?1579816772>) the variance of the FFMQ random intercept was put to 0 and the covariance between the FFMQ random intercept and IDS-SR or OQ-45 random intercept was put to zero, while the random intercept for the outcome variable was still estimated freely. This resulted in normal model convergence. This and other RI-CLSEM models showed adequate fit (Supplementary Table S8-S9) and similar results compared to the ‘traditional’ CLSEM models (Supplementary Table S10-S11).

Results from mediation analyses with the PROCESS macro (Hayes, Montoya, & Rockwood, 2017) were consistent with the SEM results: Group significantly predicted residualized change in outcome (depressive symptoms or overall functional impairment) at post-treatment, but no mediation of this effect by residualized change in mediator variables at mid-treatment (ab path) was found (Supplementary Table S12-S13).

# Supplementary Tables

Supplementary Table S1: Model fit indices for unconditional and conditional (MBCT + TAU versus TAU) linear growth models of depressive symptoms and overall functional impairment for the ITT sample.

| Model | χ^2^ (df) | CFI | TLI | RMSEA (90CI) | SRMR |
| --- | --- | --- | --- | --- | --- |
|  |  |  |  |  |  |
| Unconditional models Depressive symptoms (IDS-SR) | | | | | |
| Full sample | 2.53 (1) | .99 | .97 | .11 (.00 - .28) | .02 |
| Conditional model Depressive symptoms (IDS-SR), with Group as predictor for slope and intercept | | | | | |
| MBCT + TAU versus TAU (control) | 2.51 (2) | 1.0 | .99 | .04 (.00 - .18) | .02 |
| Conditional models Depressive symptoms (IDS-SR), with Group*moderator-variables interaction as predictor for slope and intercept | | | | | |
| Group*RRSbr | 6.27 (4) | .99 | .98 | .07 (.00 - .16) | .02 |
| Group*PTQ | 3.78 (4) | 1.0 | 1.0 | .00 (.00 - .13) | .02 |
| Group*FFMQ-SF | 7.00 (4) | .99 | .97 | .08 (.00 - .17) | .02 |
| Group*SCS-SF | 3.62 (4) | 1.0 | 1.0 | .00 (.00 - .13) | .02 |
| Group*BFT | 12.2 (4) | .96 | .87 | .14 (.05 - .23) | .03 |
|  |  |  |  |  |  |
| Unconditional models for Overall functional impairment (OQ45) | | | | | |
| Full sample ^$^ | 10.54 (2) | .94 | .92 | .18 (.08 - .29) | .09 |
|  |  |  |  |  |  |
| Conditional model Overall functional impairment (OQ45), with Group as predictor for slope and intercept | | | | | |
| MBCT + TAU versus TAU (control) ^$^ | 13.87 (3) | .95 | .90 | .16 (.08 - .26) | .09 |
|  |  |  |  |  |  |
| Conditional models Overall functional impairment (OQ45), with Group*moderator-variables interaction as predictor for slope and intercept | | | | | |
| Group*RRSbr ^$^ | 18.39 (5) | .96 | .90 | .14 (.08 - .21) | .07 |
| Group*PTQ ^$^ | 16.43 (5) | .97 | .92 | .13 (.06 - .20) | .07 |
| Group*FFMQ-SF ^$^ | 18.02 (5) | .96 | .90 | .14 (.07 - .21) | .07 |
| Group*SCS-SF ^$^ | 15.85 (5) | .97 | .92 | .13 (.06 - .20) | .07 |
| Group*BFT ^$^ | 14.44 (5) | .96 | .90 | .13 (.06 - .21) | .07 |
|  |  |  |  |  |  |
| Unconditional model Perseverative Thinking (PTQ) | | | | |  |
| Full sample ^#^ | 0.25 (2) | 1.00 | 1.00 | .00 (.00 - .08) | .02 |
|  |  |  |  |  |  |
| Conditional model Perseverative thinking (PTQ) with Group as predictor of slope and intercept | | | | | |
| MBCT + TAU versus TAU (control) ^#^ | 2.50 (3) | 1.00 | 1.00 | .00 (.00 - .14) | .03 |
|  |  |  |  |  |  |
| Unconditional model mindfulness skills (FFMQ-SF) | | | | | |
| Full sample ^#^ | 2.74 (2) | 1.00 | 0.99 | .05 (.00 - .19) | .09 |
|  |  |  |  |  |  |
| Conditional model mindfulness skills (FFMQ-SF) with Group as predictor of slope and intercept | | | | | |
| MBCT + TAU versus TAU (control) ^#^ | 3.35 (3) | 1.00 | 1.00 | .03 (.00 - .15) | .08 |
| Unconditional model Self Compassion (SCS-SF) | | | | |  |
| Full sample | 0.001 (1) | 1.00 | 1.00 | .00 (.00 - .00) | .00 |
|  |  |  |  |  |  |
| Conditional model Self Compassion (SC-SFS) with Group as predictor of slope and intercept | | | | | |
| MBCT + TAU versus TAU (control) | 0.754 (2) | 1.00 | 1.00 | .00 (.00 - .13) | .01 |
|  |  |  |  |  |  |
| Unconditional model anxiety (STAI) | | | | | |
| Full sample ^$^ | 6.48 (2) | .96 | .94 | .13 (.03 - .25) | .18 |
|  |  |  |  |  |  |
| Conditional model anxiety (STAI) with Group as predictor of slope and intercept | | | | | |
| MBCT + TAU versus TAU (control) ^$^ | 8.87 (3) | .96 | .93 | .12 (.03 - .22) | .16 |
|  |  |  |  |  |  |
| Unconditional model negative intrusive thoughts (BFT) | | | | | |
| Full sample | 0.002 (1) | 1.00 | 1.00 | .00 (.00 - .00) | .00 |
|  |  |  |  |  |  |
| Conditional model negative intrusive thoughts (BFT) with Group as predictor of slope and intercept | | | | | |
| MBCT + TAU versus TAU (control) | 0.031 (2) | 1.00 | 1.00 | .00 (.00 - .00) | .01 |
|  |  |  |  |  |  |
|  |  |  |  |  |  |
| $ For conditional and unconditional models of overall functional impairment (OQ45) and anxiety (STAI), residual variance at T1 and T2 were restricted to be equal for successful model convergence. # For the unconditional and conditional model of perseverative thinking (PTQ) and mindfulness skills (FFMQ-SF) the residual variance of T0 was put to 0 for successful model convergence.  IDS-SR = IDS-SR = Inventory of Depressive Symptomatology – Self-Report, OQ45 = Outcome Questionnaire 45, RRSbr = Brooding subscale of Ruminative Response Scale, PTQ =Perseverative Thinking Questionnaire, FFMQ-SF = Five Facet Mindfulness Questionnaire – Short-Form, SCS-SF = Self Compassion Scale – Short-Form, STAI = State-Trait Anxiety Inventory, MBCT = Mindfulness-Based Cognitive Therapy, TAU = treatment as usual, Group = MBCT + TAU versus TAU | | | | | |

Supplementary Table S2: Parameter estimates of conditional latent growth models of overall functional impairment moderated by measures of RNT, self-compassion and mindfulness skills.

| Moderator | Unstandardized Coefficient [95CI] | p-value |
| --- | --- | --- |
| RRS brooding subscale as moderator | | |
| Slope on Group | 3.53 [-4.55, 11.6] | .39 |
| Slope on RRSbr | 1.10 [0.17, 2.02] | .020 |
| Slope on Group*RRSbr | -0.66 [-1.28, -0.03] | .041 |
| *Slope on Group*RRSbr corrected for SCS-SF* | *-0.65 [-1.30, -0.00]* | ***.049*** |
| *Slope on group*RRSbr corrected for SCS-SF, Country of birth, Work situation and Current treatment* | *-0.67 [-1.34, 0.01]* | *.055* |
|  |  |  |
| PTQ as moderator | | |
| Slope on Group | 12.33 [-2.86, 27.52] | .11 |
| Slope on PTQ | 0.66 [0.01, 1.32] | .046 |
| Slope on Group*PTQ | -0.43 [-0.80, -0.07] | **.020** |
| *Slope on Group*PTQ corrected for SCS-SF* | *-0.45 [-0.82, -0.08]* | ***.017*** |
| *Slope on group*PTQ corrected for SCS-SF, Country of birth, Work situation and Current treatment* | *-0.42 [-0.80, -0.03]* | ***.034*** |
|  |  |  |
| FFMQ-SF as moderator | | |
| Slope on Group | -18.37 [-32.77, -3.97] | .012 |
| Slope on FFMQ-SF | -1.39 [-2.95, 0.18] | .082 |
| Slope on Group*FFMQ-SF | 0.92 [0.00 , 1.85] | .050 |
| *Slope on group*FFMQ corrected for, Country of birth, Work situation and Current treatment* | *0.96 [-0.08, 1.99]* | *.072* |
|  |  |  |
| SCS-SF as moderator | | |
| Slope on Group | -11.99 [-18.90, -5.08] | .001 |
| Slope on SCS-SF | -3.30 [-6.42, -0.17] | .039 |
| Slope on Group*SCS-SF | 2.37 [0.31, 4.44] | **.024** |
| *Slope on Group*SCS-SF corrected for RRSbr* | *2.25 [0.42, 4.62]* | ***.019*** |
| *Slope on group*SCS-SF corrected for RRSbr, Country of birth, Work situation and Current treatment* | *2.36 [0.01, 4.70]* | ***.049*** |
| *Slope on Group*SCS-SF corrected for PTQ* | *2.37 [0.33, 4.42]* | ***.023*** |
| *Slope on group*SCS-SF corrected for PTQ, Country of birth, Work situation and Current treatment* | *2.20 [-0.10, 4.51]* | *.061* |
|  |  |  |
| Negative intrusive thoughts (BFT) as moderator | | |
| Slope on Group | -0.24 [-4.11, 3.63] | .90 |
| Slope on BFT | 1.69 [0.07, 3.32] | .041 |
| Slope on Group*BFT | -1.46 [-2.60, -0.33] | **.012** |
| *Slope on group*BFT corrected for Country of birth, Work situation and Current treatment* | *-1.51 [-2.54, -0.48]* | ***.004*** |
|  | |  |
| IDS-SR = Inventory of Depressive Symptomatology – Self-Report, OQ-45 = Outcome Questionnaire – 45, RRSbr = Brooding subscale of Ruminative Response Scale, PTQ =Perseverative Thinking Questionnaire, FFMQ-SF = Five Facet Mindfulness Questionnaire – Short-Form, SCS-SF = Self Compassion Scale – Short-Form, STAI = State-Trait Anxiety Inventory, BFT = negative intrusive thoughts reported on the Breathing Focus Task, Group = Mindfulness-Based Cognitive Therapy (MBCT) + treatment as usual (TAU) versus TAU | | |

Supplementary Table S3: Moderation analysis of the effect of MBCT + TAU versus TAU on depressive symptoms using PROCESS in Intention To Treat Sample.

| Moderator | Coefficient (SE) | *p*-value | #BOOT Coefficient (SE) | #BOOT 95CI |
| --- | --- | --- | --- | --- |
| RRS brooding subscale as moderator (n = 113) | | | | |
| constant | 4.77 (1.16) | < .001 | 4.77 (1.17) | [2.32, 6.97] |
| Group | -6.70 (1.49) | < .001 | -6.69 (1.49) | [-9.54, -3.62] |
| RRSbr | 0.96 (0.32) | .004 | 0.95 (0.32) | [0.31, 1.57] |
| Group*RRSbr | -0.76 (0.43) | .078 | -0.75 (0.42) | [-1.59, 0.08] |
| *Group*RRSbr corrected for SCS-SF* | | *.081* | *-0.75 (0.42)* | *[-1.60, 0.08]* |
|  |  |  |  |  |
| PTQ as moderator (n = 113) | | | | |
| constant | 4.05 (1.30) | .002 | 4.05 (1.23) | [1.48, 6,34] |
| Group | -5.98 (1.62) | < .001 | -5.99 (1.56) | [-9.00, -2.87] |
| PTQ | 0.37 (0.18) | .045 | 0.37 (0.16) | [0.11, 0.78] |
| Group*PTQ | -0.43 (0.21) | .043 | -0.43 (0.20) | **[-0.86, -0.09]** |
| *Group*PTQ corrected for SCS-SF* | | *.053* | *-0.43 (0.20)* | ***[-0.85, -0.07]*** |
|  | |  |  |  |
| FFMQ-SF as moderator (n = 113) | | | | |
| constant | 4.83 (1.36) | < .001 | 4.84 (1.35) | [1.96, 7.24] |
| Group | -6.75 (1.65) | < .001 | -6.76 (1.65) | [-9.85, -3.44] |
| FFMQ-SF | -1.02 (0.53) | .058 | -1.03 (0.52) | [-2.00, 0.01] |
| Group*FFMQ-SF | 0.90 (0.65) | .17 | 0.92 (0.63) | [-0.35, 2.14] |
|  |  |  |  |  |
| SCS-SF as moderator (n = 113 ) | | | | |
| constant | 4.68 (1.19) | < .001 | 4.65 (1.18) | [2.24, 6.83] |
| Group | -6.57 (1.53) | < .001 | -6.53 (1.51) | [-9.54, -3.61] |
| SCS-SF | -3.11 (1.04) | .003 | -3.09 (0.94) | [-5.09, -1.32] |
| Group*SCS-SF | 3.43 (1.49) | .023 | 3.50 (1.33) | **[0.89, 6.12]** |
| *Group*SCC-SF corrected for RRSbr* | | *.016* | *3.67 (1.35)* | ***[1.06, 6.34]*** |
| *Group*SCS-SF corrected for PTQ* | | *.024* | *3.52 (1.35)* | ***[0.80, 6,07]*** |
|  | | | | |
| Negative intrusive thoughts (BFT) as moderator (n = 95) | | | | |
| constant | 4.07 (1.43) | .006 | 3.98 (1.41) | [0.93, 6.48] |
| Group | -6.41 (1.81) | < .001 | -6.31 (1.79) | [-9.79, -2.77] |
| BFT | 0.65 (0.56) | .25 | 0.63 (0.53) | [-0.48, 1.61] |
| Group*BFT | -1.31 (0.77) | .09 | -1.29 (0.74) | [-2.71, 0.18] |
|  | | | | |
| *Note.* A heteroscedasticity consistent standard error and covariance matrix estimator was used to estimate model parameters in a robust manner.  # Number of bootstraps for bias-corrected bootstrap confidence intervals = 5000  IDS-SR = IDS-SR = Inventory of Depressive Symptomatology – Self-Report, OQ45 = Outcome Questionnaire 45, RRSbr = Brooding subscale of Ruminative Response Scale, PTQ =Perseverative Thinking Questionnaire, FFMQ-SF = Five Facet Mindfulness Questionnaire – Short-Form, SCS-SF = Self Compassion Scale – Short-Form, Group = Mindfulness-Based Cognitive Therapy (MBCT) + treatment as usual (TAU) versus TAU | | | | |

Supplementary Table S4: Moderation analysis of the effect of MBCT + TAU versus TAU on overall functional impairment using PROCESS in Intention To Treat Sample.

| Moderator | Coefficient (SE) | *p*-value | ^#^BOOT Coefficient (SE) | ^#^BOOT 95CI |
| --- | --- | --- | --- | --- |
| RRS brooding subscale as moderator (n = 113) | | | | |
| constant | 6.65 (1.90) | .001 | 6.62 (1.91) | [2.41, 9.94] |
| Group | -9.34 (2.56) | < .001 | -9.21 (2.60) | [-14.25, -4.13] |
| RRSbr | 1.16 (0.46) | .013 | 1.38 (0.59) | [0.27, 2.18] |
| Group*RRSbr | -105 (0.71) | .14 | -1.42 (0.81) | [-2.44, 0.37] |
|  |  |  |  |  |
| PTQ as moderator (n = 113) | | | | |
| constant | 5.62 (2.44) | .02 | 5.57 (2.11) | [1.22, 9.46] |
| Group | -8.41 (3.00) | .001 | -8.41 (2.76) | [-13.81, -2.93] |
| PTQ | 0.61 (0.41) | .15 | 0.62 (0.35) | [0.02, 1.40] |
| Group*PTQ | -0.85 (0.46) | .07 | -0.87 (0.40) | **[-1.72, -0.14]** |
| *Group*PTQ corrected for SCS-SF* | | *.07* | *-0.87 (0.41)* | ***[-1.72, -0.11]*** |
|  | |  |  |  |
| FFMQ-SF as moderator (n = 113) | | | | |
| constant | 6.78 (2.01) | .001 | 6.75 (2.01) | [2.08, 10.05] |
| Group | -9.41 (2.63) | .001 | -9.39 (2.67) | [-14.51, -3.98] |
| FFMQ-SF | -1.36 (0.80) | .09 | -1.35 (0.78) | [-2.86, 0.23] |
| Group*FFMQ-SF | 1.65 (0.98) | .10 | 1.64 (0.98) | [-0.38, 3.53] |
|  |  |  |  |  |
| SCS-SF as moderator (n = 113 ) | | | | |
| constant | 6.52 (1.93) | .001 | 6.47 (1.91) | [2.23, 9.81] |
| Group | -9.15 (2.58) | .001 | -9.13 (2.59) | [-14.10, -3.96] |
| SCS-SF | -3.41 (1.51) | .03 | -3.43 (1.43) | [-6.39, -0.74] |
| Group*SCS-SF | 4.62 (2.21) | .04 | 4.72 (2.15) | **[0.33, 8.84]** |
| *Group*SCS-SF corrected for RRSbr* | | *.03* | *4.96 (2.17)* | ***[0.65, 9.04]*** |
| *Group*SCS-SF corrected for PTQ* | | *.04* | *4.77 (2.17)* | ***[0.15, 8.79]*** |
|  | | | | |
| Negative intrusive thoughts (BFT) as moderator (n = 95) | | | | |
| constant | 5.04 (2.60) | .06 | 4.91 (2.57) | [-0.44, 9.66] |
| Group | -7.70 (3.24) | .02 | -7.60 (3.18) | [-13.94, -1.34] |
| BFT | 0.76 (0.82) | .36 | 0.74 (0.76) | [-0.68, 2.24] |
| Group*BFT | -2.92 (1.29) | .03 | -2.93 (1.23) | **[-5.27, -0.52]** |
|  | | | | |
| *Note.* A heteroscedasticity consistent standard error and covariance matrix estimator was used to estimate model parameters in a robust manner.  # Number of bootstraps for bias-corrected bootstrap confidence intervals = 5000  IDS-SR = IDS-SR = Inventory of Depressive Symptomatology – Self-Report, OQ45 = Outcome Questionnaire 45, RRSbr = Brooding subscale of Ruminative Response Scale, PTQ =Perseverative Thinking Questionnaire, FFMQ-SF = Five Facet Mindfulness Questionnaire – Short-Form, SCS-SF = Self Compassion Scale – Short-Form, Group = Mindfulness-Based Cognitive Therapy (MBCT) + treatment as usual (TAU) versus TAU | | | | |

Supplementary Table S5: Fit statistics for the cross-lagged structural equation models with depressive symptoms (IDS-SR) as outcome.

| Model | χ^2^ (df) | CFI | TLI | RMSEA (90CI) | SRMR |
| --- | --- | --- | --- | --- | --- |
| RRSbr as mediator | 5.50 (4) | 1.00 | .98 | .06 (.00 - .16) | .02 |
| PTQ as mediator | 5.78 (4) | 1.00 | .98 | .06 (.00 - .16) | .02 |
| FFMQ-SF as mediator | 4.94 (4) | 1.00 | .99 | .04 (.00 - .15) | .02 |
| SCS-SF as mediator | 6.06 (4) | 1.00 | .97 | .07 (.00 - .16) | .02 |
| BFT as mediator | 5.51 (4) | .99 | .97 | .06 (.00 - .16) | .03 |
|  | | | | | |
| IDS-SR = Inventory of Depressive Symptomatology – Self-Report, OQ-45 = Outcome Questionnaire – 45, RRSbr = Brooding subscale of Ruminative Response Scale, PTQ =Perseverative Thinking Questionnaire, FFMQ-SF = Five Facet Mindfulness Questionnaire – Short-Form, SCS-SF = Self Compassion Scale – Short-Form, STAI = State-Trait Anxiety Inventory, BFT = negative intrusive thoughts reported on the Breathing Focus Task, CFI = comparative fit index, TLI = Tucker-Lewis index, RMSEA = root mean square error of approximation, SRMR = Standardized Root Mean Square Residual | | | | | |

Supplementary Table S6: Fit statistics for the cross-lagged structural equation models with overall functional impairment (OQ45) as outcome.

| Model | χ^2^ (df) | CFI | TLI | RMSEA (90CI) | SRMR |
| --- | --- | --- | --- | --- | --- |
| RRSbr as mediator | 2.82 (4) | 1.00 | 1.00 | .00 (.00 - .12) | .01 |
| PTQ as mediator | 4.27 (4) | 1.00 | 1.00 | .02 (.00 - .14) | .02 |
| FFMQ-SF as mediator | 3.08 (4) | 1.00 | 1.00 | .00 (.00 - .12) | .01 |
| SCS-SF as mediator | 3.85 (4) | 1.00 | 1.00 | .00 (.00 - .13) | .01 |
| BFT as mediator | 7.20 (4) | .99 | .94 | .08 (.00 - .18) | .03 |
|  |  |  |  |  |  |
| IDS-SR = IDS-SR = Inventory of Depressive Symptomatology – Self-Report, OQ45 = Outcome Questionnaire 45, RRSbr = Brooding subscale of Ruminative Response Scale, PTQ =Perseverative Thinking Questionnaire, FFMQ-SF = Five Facet Mindfulness Questionnaire – Short-Form, SCS-SF = Self Compassion Scale – Short-Form, BFT = negative intrusive thoughts reported on the Breathing Focus Task, | | | | | |

Supplementary Table S7: Cross-lagged structural equation model output for the mediation pathways with overall functional impairment (OQ45) at post-treatment (T2) as outcome.

| Mediator | Predictor | Outcome | Path | β | 95%CI | *p* |
| --- | --- | --- | --- | --- | --- | --- |
| RRSbr | Group | RRSbr T1 | a | -0.08 | [-0.21, 0.05] | .20 |
|  | RRSbr T1 | OQ45 T2 | b | 0.12 | [0.00, 0.24] | .045 |
|  | Group | OQ45 T2 | **c** | **-0.13** | **[-0.21, -0.04]** | **.003** |
|  | Group via RRSbr T1 | OQ45 T2 | ab | -0.01 | [-0.03, 0.01] | .29 |
| PTQ | Group | PTQ T1 | a | 0.03 | [-0.07, 0.13] | .56 |
|  | PTQ T1 | OQ45 T2 | b | -0.02 | [-0.17, 0.13] | .78 |
|  | Group | OQ45 T2 | **c** | **-0.12** | **[-0.20, -0.04]** | **.002** |
|  | Group via PTQ T1 | OQ45 T2 | ab | -0.00 | [-0.01, 0.01] | .81 |
| FFMQ | Group | FFMQ T1 | a | 0.03 | [-0.08, 0.15] | .58 |
|  | FFMQ T1 | OQ45 T2 | b | -0.06 | [-0.17, 0.05] | .31 |
|  | Group | OQ45 T2 | **c** | **-0.13** | **[-0.21, -0.05]** | **.002** |
|  | Group via FFMQ T1 | OQ45 T2 | ab | -0.00 | [-0.01, 0.01] | .64 |
| SCS | Group | SCS T1 | a | 0.04 | [-0.07, 0.14] | .49 |
|  | SCS T1 | OQ45 T2 | b | 0.00 | [-0.13, 0.13] | 1.00 |
|  | Group | OQ45 T2 | **c** | **-0.12** | **[-0.21, -0.04]** | **.005** |
|  | Group via SCS T1 | OQ45 T2 | ab | 0.00 | [-0.01, 0.01] | 1.00 |
| BFT | Group | BFT T1 | a | 0.05 | [-0.15, 0.26] | .61 |
|  | BFT T1 | OQ45 T2 | b | -0.01 | [-0.09, 0.06] | .72 |
|  | Group | OQ45 T2 | **c** | **-0.12** | **[-0.20, -0.04]** | **.003** |
|  | Group via BFT T1 | OQ45 T2 | ab | -0.00 | [-0.01, 0.00] | .77 |
|  | | | | | | |
| IDS-SR = IDS-SR = Inventory of Depressive Symptomatology – Self-Report, OQ45 = Outcome Questionnaire 45, RRSbr = Brooding subscale of Ruminative Response Scale, PTQ =Perseverative Thinking Questionnaire, SCS-SF = Self Compassion Scale – Short-Form, FFMQ-SF = Five Facet Mindfulness Questionnaire – Short-Form, BFT = negative intrusive thoughts reported on the Breathing Focus Task, Group = Mindfulness-Based Cognitive Therapy (MBCT) + treatment as usual (TAU) versus TAU | | | | | | |

Supplementary Table S8: Fit statistics for the random-intercept cross-lagged structural equation models with depressive symptoms (IDS-SR) as outcome.

| Model | χ^2^ (df) | CFI | TLI | RMSEA (90CI) | SRMR |
| --- | --- | --- | --- | --- | --- |
| RRSbr as mediator | 0.44 (1) | 1.00 | 1.00 | .00 (.00 - .14) | .00 |
| PTQ as mediator | 0.33 (1) | 1.00 | 1.00 | .00 (.00 - .20) | .01 |
| FFMQ-SF as mediator | 5.04 (3) | 1.00 | 0.97 | .08 (.00 - .18) | .02 |
| SCS-SF as mediator | 0.84 (1) | 1.00 | 1.00 | .00 (.00 - .23) | .01 |
| BFT as mediator | 1.87 (1) | 1.00 | .92 | .09 (.00 - .27) | .02 |
|  |  |  |  |  |  |
| IDS-SR = IDS-SR = Inventory of Depressive Symptomatology – Self-Report, OQ45 = Outcome Questionnaire 45, RRSbr = Brooding subscale of Ruminative Response Scale, PTQ =Perseverative Thinking Questionnaire, SCS-SF = Self Compassion Scale – Short-Form, FFMQ-SF = Five Facet Mindfulness Questionnaire – Short-Form, BFT = negative intrusive thoughts reported on the Breathing Focus Task | | | | | |

Supplementary Table S9: Fit statistics for the random-intercept cross-lagged structural equation models with overall functional impairment (OQ45) as outcome.

| Model | χ^2^ (df) | CFI | TLI | RMSEA (90CI) | SRMR |
| --- | --- | --- | --- | --- | --- |
| RRSbr as mediator | 0.62 (1) | 1.00 | 1.00 | .00 (.00 - .22) | .01 |
| PTQ as mediator | .25 (1) | 1.00 | 1.00 | .00 (.00 - .19) | .00 |
| FFMQ-SF as mediator | 2.84 (3) | 1.00 | 1.00 | .00 (.00 - .15) | .01 |
| SCS-SF as mediator | .00 (1) | 1.00 | 1.00 | .00 (.00 - .00) | .00 |
| BFT as mediator | 1.26 (1) | 1.00 | 0.98 | .05 (.00 - .25) | .02 |
|  |  |  |  |  |  |
| IDS-SR = IDS-SR = Inventory of Depressive Symptomatology – Self-Report, OQ45 = Outcome Questionnaire 45, RRSbr = Brooding subscale of Ruminative Response Scale, PTQ =Perseverative Thinking Questionnaire, SCS-SF = Self Compassion Scale – Short-Form, FFMQ-SF = Five Facet Mindfulness Questionnaire – Short-Form, BFT = negative intrusive thoughts reported on the Breathing Focus Task | | | | | |

Supplementary Table S10: Random-intercept cross-lagged structural equation model output for the mediation pathways with depressive symptoms (IDS-SR) at post-treatment (T2) as outcome.

| Mediator | Predictor | Outcome | Path | β | 95%CI | *p* |
| --- | --- | --- | --- | --- | --- | --- |
| RRSbr | Group | RRSbr T1 | a | -0.09 | [-0.36, 0.18] | .49 |
|  | RRSbr T1 | IDS-SR T2 | b | -0.01 | [-0.31, 0.28] | .93 |
|  | Group | IDS-SR T2 | c | **-0.33** | **[-0.56, -0.10]** | **.005** |
|  | Group via RRSbr T1 | IDS-SR T2 | ab | .00 | [-0.03, 0.03] | .93 |
| PTQ | Group | PTQ T1 | a | 0.03 | [-0.10, 0.16] | .64 |
|  | PTQ T1 | IDS-SR T2 | b | -0.03 | [-0.37, 0.31] | .86 |
|  | Group | IDS-SR T2 | c | **-0.32** | **[-0.54, -0.09]** | **.006** |
|  | Group via PTQ T1 | IDS-SR T2 | ab | -0.00 | [-0.01, 0.01] | .87 |
| FFMQ-SF | Group | FFMQ T1 | a | 0.04 | [-0.09, 0.17] | .53 |
|  | FFMQ T1 | IDS-SR T2 | b | -0.09 | [-0.44, 0.26] | .60 |
|  | Group | IDS-SR T2 | c | **-0.23** | **[-0.41, -0.05]** | **.012** |
|  | Group via FFMQ T1 | IDS-SR T2 | ab | -0.04 | [-0.02, 0.01] | 0.65 |
| SCS-SF | Group | SCS T1 | a | 0.06 | [-0.11, 0.23] | .50 |
|  | SCS T1 | IDS-SR T2 | b | -0.09 | [-0.39, 0.21] | .55 |
|  | Group | IDS-SR T2 | c | **-0.34** | **[-0.58, -0.09]** | **.007** |
|  | Group via SCS T1 | IDS-SR T2 | ab | -0.01 | [-0.03, 0.02] | .65 |
| BFT | Group | BFT T1 | a | 0.03 | [-0.22, 0.28] | .81 |
|  | BFT T1 | IDS-SR T2 | b | -0.06 | [-0.33, 0.21] | .66 |
|  | Group | IDS-SR T2 | c | **-0.33** | **[-0.58, -0.08]** | **.010** |
|  | Group via BFT T1 | IDS-SR T2 | ab | -0.00 | [-0.02, 0.01] | .80 |
|  |  |  |  |  |  |  |
| IDS-SR = IDS-SR = Inventory of Depressive Symptomatology – Self-Report, OQ45 = Outcome Questionnaire 45, RRSbr = Brooding subscale of Ruminative Response Scale, PTQ =Perseverative Thinking Questionnaire, SCS-SF = Self Compassion Scale – Short-Form, FFMQ-SF = Five Facet Mindfulness Questionnaire – Short-Form, BFT = negative intrusive thoughts reported on the Breathing Focus Task, Group = Mindfulness-Based Cognitive Therapy (MBCT) + treatment as usual (TAU) versus TAU | | | | | | |

Supplementary Table S11: Random-intercept cross-lagged structural equation model output for the mediation pathways with overall functional impairment (OQ45) at post-treatment (T2) as outcome.

| Mediator | Predictor | Outcome | Path | β | 95%CI | *p* |
| --- | --- | --- | --- | --- | --- | --- |
| RRSbr | Group | RRSbr T1 | a | -0.08 | [-0.32, 0.16] | .52 |
|  | RRSbr T1 | OQ45 T2 | b | 0.06 | [-0.17, 0.30] | .59 |
|  | Group | OQ45 T2 | **c** | **-0.18** | **[-0.31, -0.05]** | **.007** |
|  | Group via RRSbr T1 | OQ45 T2 | ab | -0.01 | [-0.03, 0.02] | .66 |
| PTQ | Group | PTQ T1 | a | 0.03 | [-0.09, 0.15] | .60 |
|  | PTQ T1 | OQ45 T2 | b | -0.03 | [-0.23, 0.18] | .79 |
|  | Group | OQ45 T2 | **c** | **-0.17** | **[-0.29, -0.05]** | **.008** |
|  | Group via PTQ T1 | OQ45 T2 | ab | -0.00 | [-0.01, 0.01] | .92 |
| FFMQ-SF | Group | FFMQ T1 | a | 0.03 | [-0.09, 0.15] | 0.65 |
|  | FFMQ T1 | OQ45 T2 | b | -0.08 | [-0.24, 0.08] | 0.30 |
|  | Group | OQ45 T2 | c | **-0.14** | **[-0.23, -0.05]** | **0.003** |
|  | Group via FFMQ T1 | OQ45 T2 | ab | -0.00 | [-0.01, 0.01] | 0.67 |
| SCS-SF | Group | SCS T1 | a | 0.02 | [-0.16, 0.19] | .87 |
|  | SCS T1 | OQ45 T2 | b | 0.02 | [-0.24, 0.29] | .86 |
|  | Group | OQ45 T2 | **c** | **-0.17** | **[-0.31, 0.03]** | **0.02** |
|  | Group via SCS T1 | OQ45 T2 | ab | 0.00 | [-0.01, 0.01] | .90 |
| BFT | Group | BFT T1 | a | 0.03 | [-0.21, 0.28] | 0.80 |
|  | BFT T1 | OQ45 T2 | b | 0.04 | [-0.12, 0.19] | 0.63 |
|  | Group | OQ45 T2 | **c** | **-0.19** | **[-0.32, -0.47]** | **0.009** |
|  | Group via BFT T1 | OQ45 T2 | ab | 0.00 | [-0.01, 0.01] | 0.82 |
|  |  |  |  |  |  |  |
| IDS-SR = IDS-SR = Inventory of Depressive Symptomatology – Self-Report, OQ45 = Outcome Questionnaire 45, RRSbr = Brooding subscale of Ruminative Response Scale, PTQ =Perseverative Thinking Questionnaire, SCS-SF = Self Compassion Scale – Short-Form, FFMQ-SF = Five Facet Mindfulness Questionnaire – Short-Form, BFT = negative intrusive thoughts reported on the Breathing Focus Task, Group = Mindfulness-Based Cognitive Therapy (MBCT) + treatment as usual (TAU) versus TAU | | | | | | |

Supplementary Table S12: Mediation analyses using PROCESS with depressive symptoms as outcome.

| Mediator | Predictor | Outcome | Path | B [95CI] | *β ^#^* |
| --- | --- | --- | --- | --- | --- |
| RRSbr | Group | rcs RRSbr (T1T0) | a | -0.38 [-1.27, 0.50] | -0.18 |
| N = 108 | rcs RRSbr (T1T0) | rcs IDS-SR (T2T0) | **b** | **0.93 [0.21, 1.66]** | **0.23** |
|  | Group | rcs IDS-SR (T2T0) | **c** | **-6.07 [-9.41, -2.73]** | **-0.69** |
|  | Group via rcs RRSbr (T1T0) | rcs IDS-SR (T2T0) | ab | -0.36 [-1.5, 0.41] | -0.04 [-0.16, 0.05] |
| PTQ | Group | rcs PTQ (T1T0) | a | -0.11 [-3.03, 2.81] | -0.02 |
| N = 108 | rcs PTQ (T1T0) | rcs IDS-SR (T2T0) | **b** | **0.24 [0.03, 0.47]** | **0.20** |
|  | Group | rcs IDS-SR (T2T0) | **c** | **-6.40 [-9.75, -3.06]** | **-0.73** |
|  | Group via rcs PTQ (T1T0) | rcs IDS-SR (T2T0) | ab | -0.03 [-1.04, 0.70] | -0.00 [-0.12, 0.08] |
| FFMQ-SF | Group | rcs FFMQ (T1T0) | a | 0.36 [-0.36, 1.08] | 0.21 |
| N = 108 | rcs FFMQ (T1T0) | rcs IDS-SR (T2T0) | **b** | **-1.19 [-2.08, -0.30]** | **-0.24** |
|  | Group | rcs IDS-SR (T2T0) | **c** | **-6.00 [-9.34, -2.67]** | **-0.69** |
|  | Group via rcs FFMQ (T1T0) | rcs IDS-SR (T2T0) | ab | -0.43 [-1.42, 0.40] | -0.05 [-0.16, 0.05] |
| SCS-SF | Group | rcs SCS (T1T0) | a | 0.10 [-0.17, 0.37] | 0.15 |
| N = 108 | rcs SCS (T1T0) | rcs IDS-SR (T2T0) | **b** | **-4.05 [-6.36, -1.75]** | **-0.30** |
|  | Group | rcs IDS-SR (T2T0) | **c** | **-6.03 [-9.28, -2.78]** | **-0.69** |
|  | Group via rcs SCS (T1T0) | rcs IDS-SR (T2T0) | ab | -0.41 [-1.56, 0.55] | -0.05 [-0.17, 0.07] |
| BFT | Group | rcs BFT (T1T0) | a | -0.37 [-1.37, 0.64] | -0.18 |
| N = 83 | rcs BFT (T1T0) | rcs IDS-SR (T2T0) | **b** | **0.48 [-0.41, 1.37]** | **0.11** |
|  | Group | rcs IDS-SR (T2T0) | **c** | **-5.84 [-9.91, -1.77]** | **-0.66** |
|  | Group via rcs BFT (T1T0) | rcs IDS-SR (T2T0) | ab | -0.18 [-1.34, 0.42] | -0.02 [-0.15, 0.05] |
|  |  |  |  |  |  |
| rcs = residualized change score, RRSbr = brooding subscale of ruminative response scale, PTQ = perseverative thinking questionnaire, SCS = self-compassion scale, FFMQ = five-facet mindfulness questionnaire, BFT = negative intrusive thoughts reported on the Breathing Focus Task, Group = Mindfulness-Based Cognitive Therapy (MBCT) + treatment as usual (TAU) versus TAU  # PROCESS in R does not provide 95% CI’s of the standardized regression coefficients, but does only so for the indirect (mediation) path. | | | | | |

Supplementary Table S13: Mediation analyses using PROCESS with overall functional impairment as outcome.

| Mediator | Predictor | Outcome | Path | B [95CI] | *β ^#^* |
| --- | --- | --- | --- | --- | --- |
| RRSbr | Group | rcs RRSbr (T1T0) | a | -0.39 [-1.27, 0.50] | -0.18 |
| N = 108 | rcs RRSbr (T1T0) | rcs OQ45 (T2T0) | **b** | **1.51 [0.22, 2.79]** | **.21** |
|  | Group | rcs OQ45 (T2T0) | **c** | **-8.63 [-14.5, -2.72]** | **-0.57** |
|  | Group via rcs RRSbr (T1T0) | rcs OQ45 (T2T0) | ab | -0.58 [-2.28, 0.75] | -0.04 [-0.15, 0.05] |
| PTQ | Group | rcs PTQ (T1T0) | a | -0.11 [-3.03, 2.81] | -0.02 |
| N = 108 | rcs PTQ (T1T0) | rcs OQ45 (T2T0) | **b** | **0.48 [0.09, 0.86]** | **0.22** |
|  | Group | rcs OQ45 (T2T0) | **c** | **-9.16 [-15.0, -3.29]** | **-0.61** |
|  | Group via rcs PTQ (T1T0) | rcs OQ45 (T2T0) | ab | -0.05 [-1.84, 1.22] | -0.00 [-0.12, 0.08] |
| FFMQ | Group | rcs FFMQ (T1T0) | a | 0.36 [-0.35, 1.08] | 0.21 |
| N = 108 | rcs FFMQ (T1T0) | rcs OQ45 (T2T0) | **b** | **-2.62 [-4.16, -1.08]** | **-0.30** |
|  | Group | rcs OQ45 (T2T0) | **c** | **-8.27 [-14.0, -2.51]** | **-0.55** |
|  | Group via rcs FFMQ (T1T0) | rcs OQ45 (T2T0) | ab | -0.94 [-3.01, 0.98] | -0.06 [-0.20, 0.06] |
| SCS | Group | rcs SCS (T1T0) | a | 0.10 [-0.17, 0.37] | 0.15 |
| N = 108 | rcs SCS (T1T0) | rcs OQ45 (T2T0) | **b** | **-8.30 [-12.3, -4.32]** | **-0.36** |
|  | Group | rcs OQ45 (T2T0) | **c** | **-8.38 [-14.0, -2.77]** | **-0.56** |
|  | Group via rcs SCS (T1T0) | rcs OQ45 (T2T0) | ab | -0.83 [-3.17, 1.24] | -0.06 [-0.20, 0.08] |
| BFT | Group | rcs BFT (T1T0) | a | -0.37 [-1.37, 0.64] | -0.18 |
| N = 83 | rcs BFT (T1T0) | rcs OQ45 (T2T0) | b | 1.25 [-0.25, 2.74] | 0.18 |
|  | Group | rcs OQ45 (T2T0) | c | -6.80 [-13.6, 0.03] | -0.47 |
|  | Group via rcs BFT (T1T0) | rcs OQ45 (T2T0) | ab | -0.46 [-2.58, 0.79] | -0.03 [-0.17, 0.05] |
|  |  |  |  |  |  |
| rcs = residualized change score, RRSbr = brooding subscale of ruminative response scale, PTQ = perseverative thinking questionnaire, SCS = self-compassion scale, FFMQ = five-facet mindfulness questionnaire, BFT = negative intrusive thoughts reported on the Breathing Focus Task, Group = Mindfulness-Based Cognitive Therapy (MBCT) + treatment as usual (TAU) versus TAU  # PROCESS in R does not provide 95% CI’s of the standardized regression coefficients, but does only so for the indirect (mediation) path. | | | | | |

# Supplementary Figures


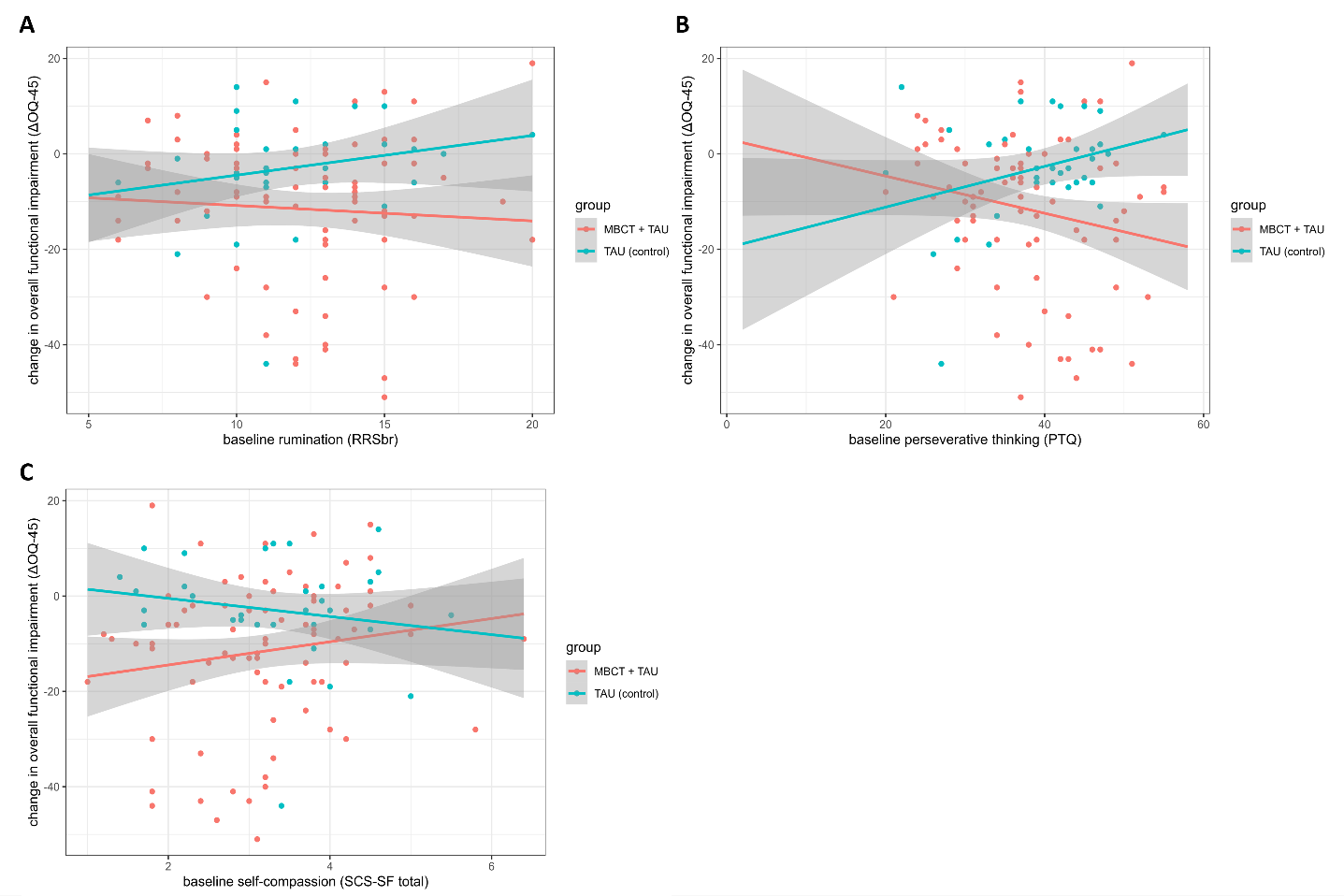


Supplementary Figure S1: Moderation of the effect of Group on rate of change in overall functional impairment by pre-treatment levels of rumination, perseverative thinking and self-compassion. This figure displays change in overall functional impairment from pre- to post-treatment in relation to pre-treatment levels of A) rumination, B) perseverative thinking, and C) self-compassion. Plots display scores for individual participants with a least square regression line (and 95% confidence level) for patients within the MBCT + TAU (red) and control (TAU; blue) condition. MBCT = Mindfulness-Based Cognitive Therapy, TAU = treatment us usual.


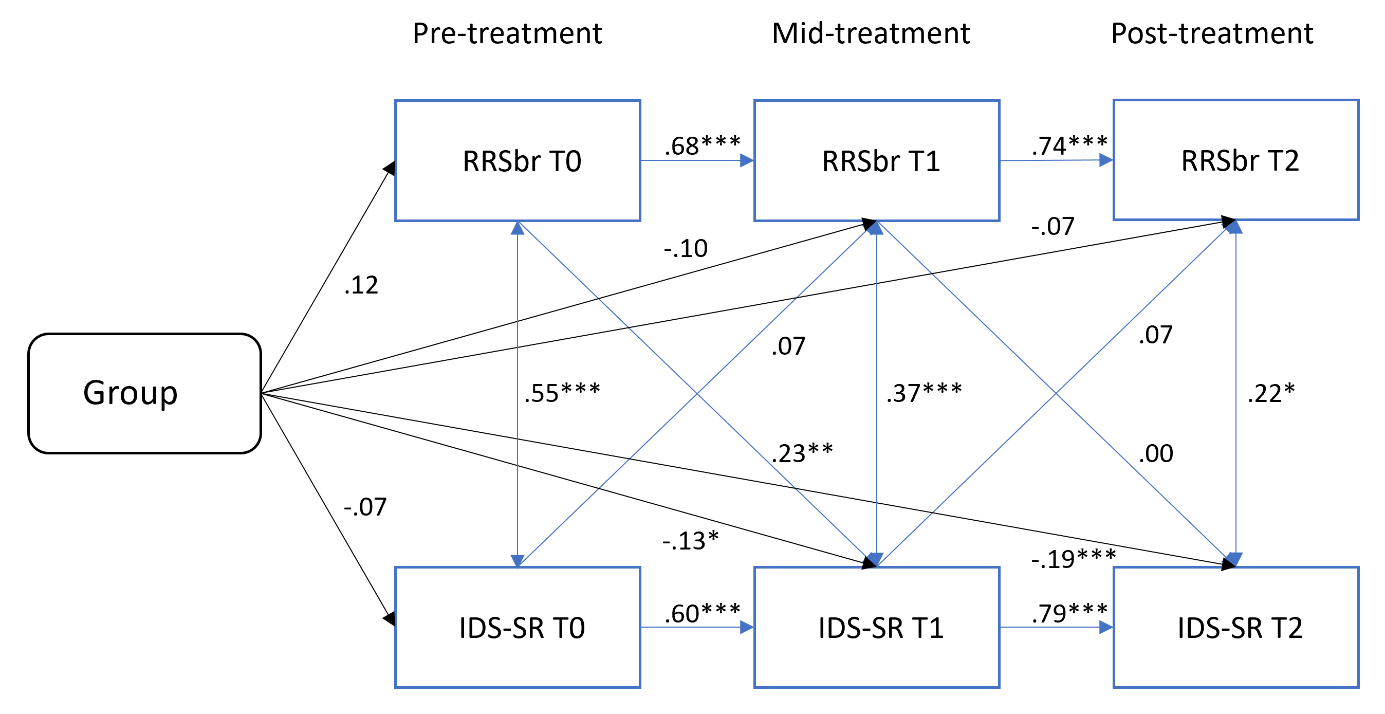


Supplementary Figure S2: Cross-lagged structural equation panel model for mediation of depressive symptoms at post-treatment (T2) by rumination at mid-treatment (T1). Group = Mindfulness-Based Cognitive Therapy (MBCT) + Treatment as Usual (TAU) versus TAU, IDS-SR = Inventory of Depressive Symptomatology – Self-report, RRSbr = Brooding subscale of Ruminative Response Scale.


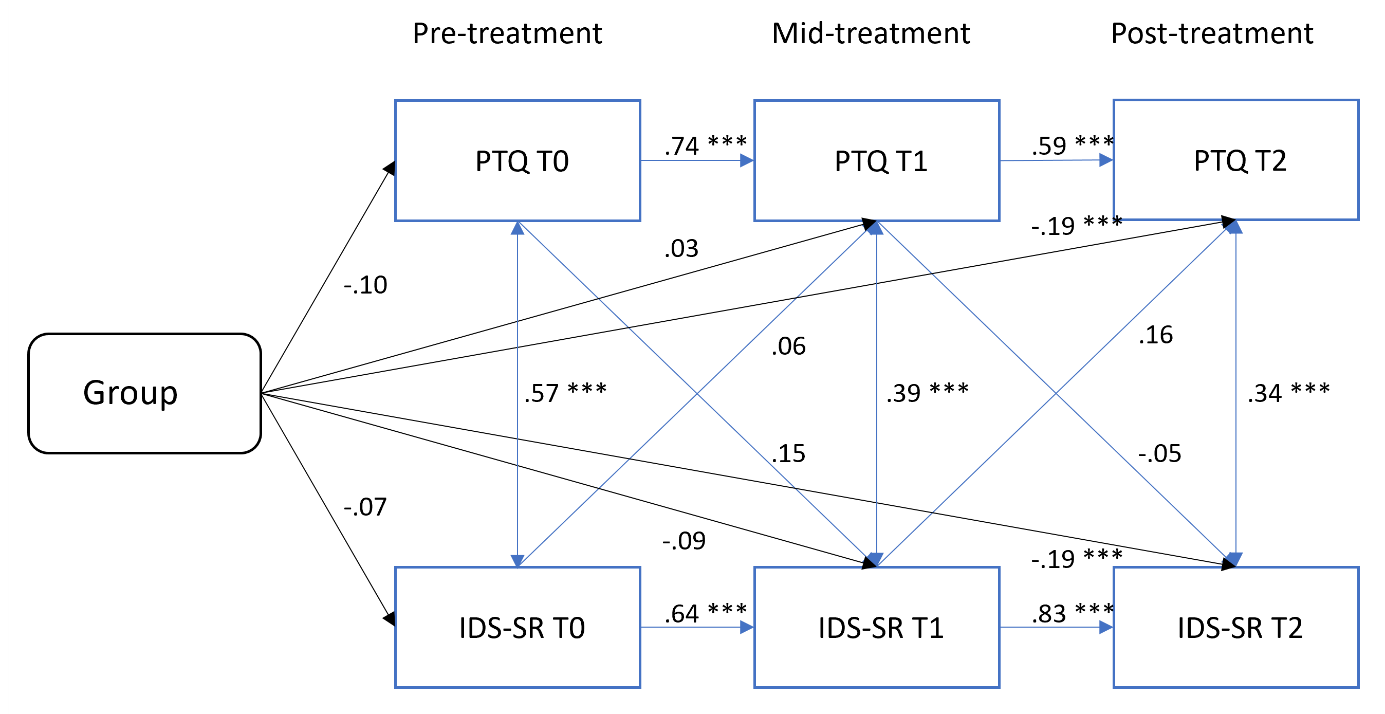


Supplementary Figure S3: Cross-lagged structural equation panel model for mediation of depressive symptoms at post-treatment (T2) by perseverative thinking at mid-treatment (T1). Group = Mindfulness-Based Cognitive Therapy (MBCT) + Treatment as Usual (TAU) versus TAU, IDS-SR = Inventory of Depressive Symptomatology – Self-report, PTQ = Perseverative Thinking Questionnaire.


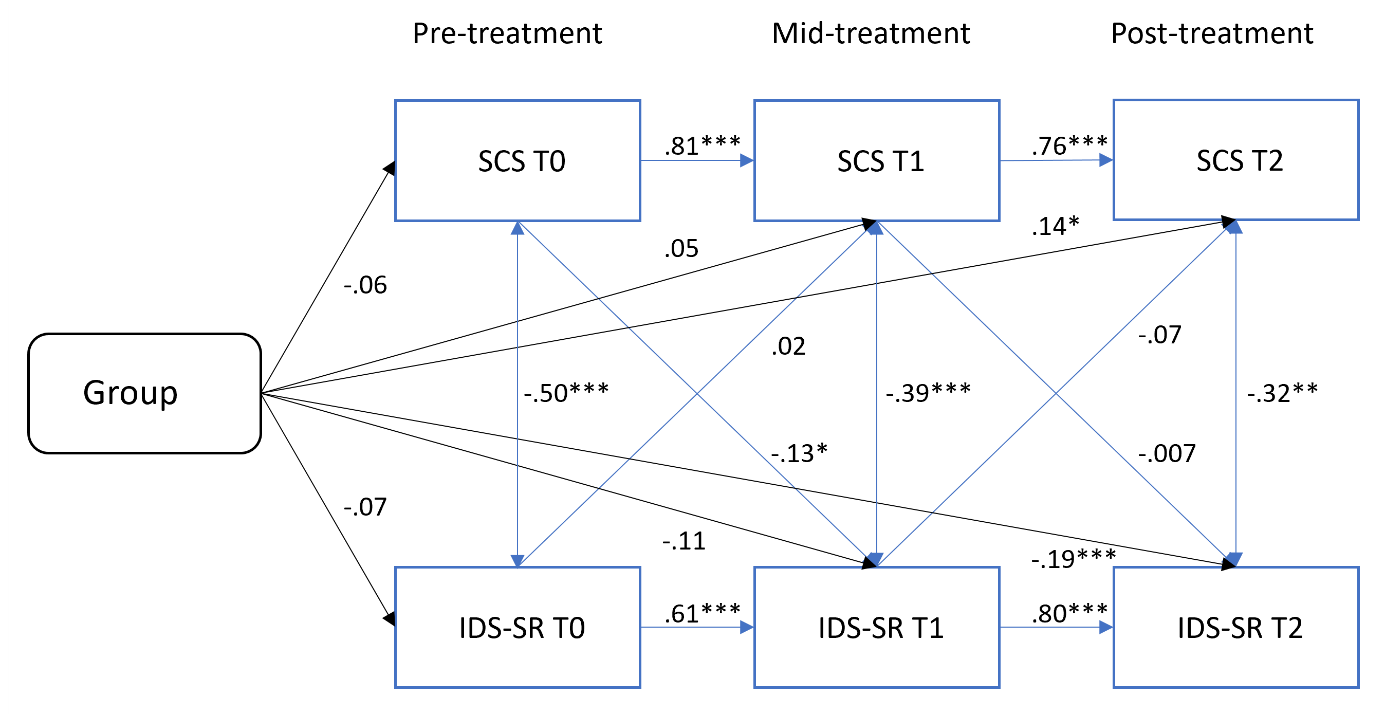


Supplementary Figure S4: Cross-lagged structural equation panel model for mediation of depressive symptoms at post-treatment (T2) by self-compassion at mid-treatment (T1). Group = Mindfulness-Based Cognitive Therapy (MBCT) + Treatment as Usual (TAU) versus TAU, IDS-SR = Inventory of Depressive Symptomatology – Self-report, SCS = Self-compassion Scale – Short-Form.


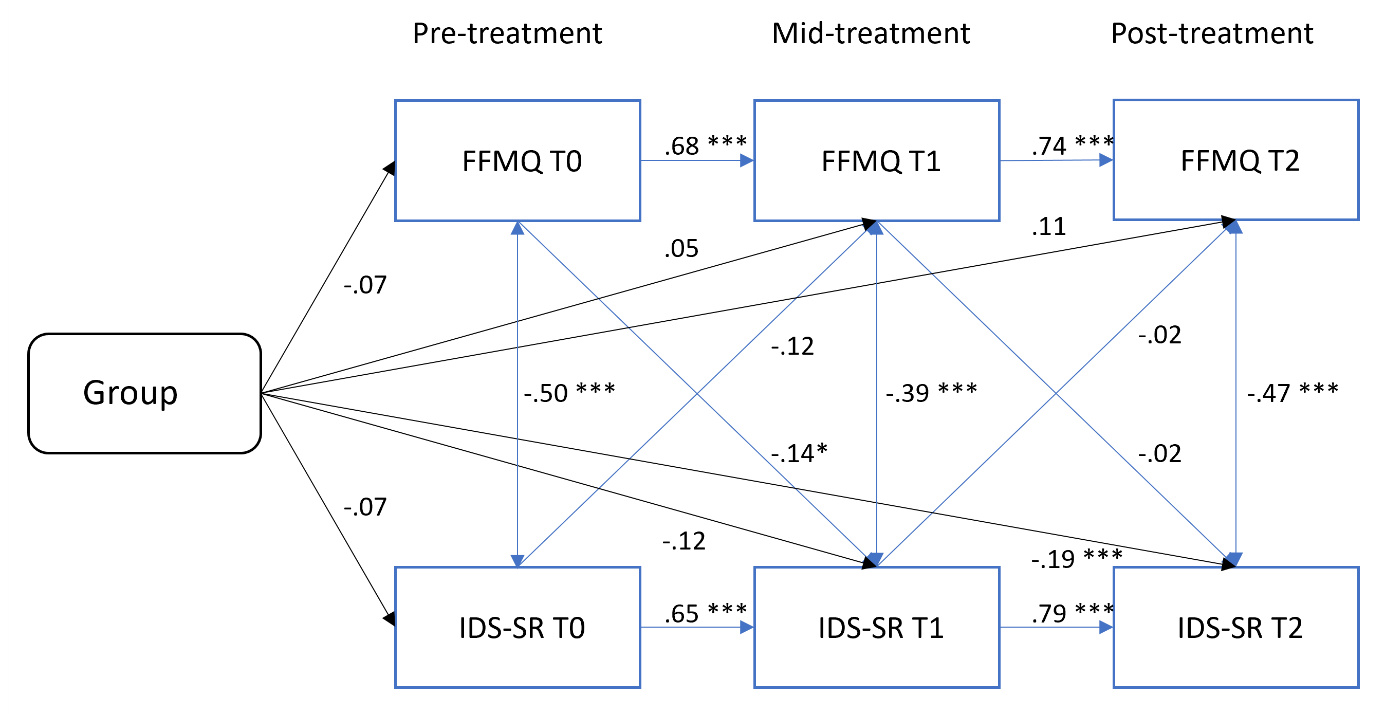


Supplementary Figure S5: Cross-lagged structural equation panel model for mediation of depressive symptoms at post-treatment (T2) by mindfulness skills at mid-treatment (T1). Group = Mindfulness-Based Cognitive Therapy (MBCT) + Treatment as Usual (TAU) versus TAU, IDS-SR = Inventory of Depressive Symptomatology – Self-report, FFMQ = Five Facet Mindfulness Questionnaire – Sort-Form.


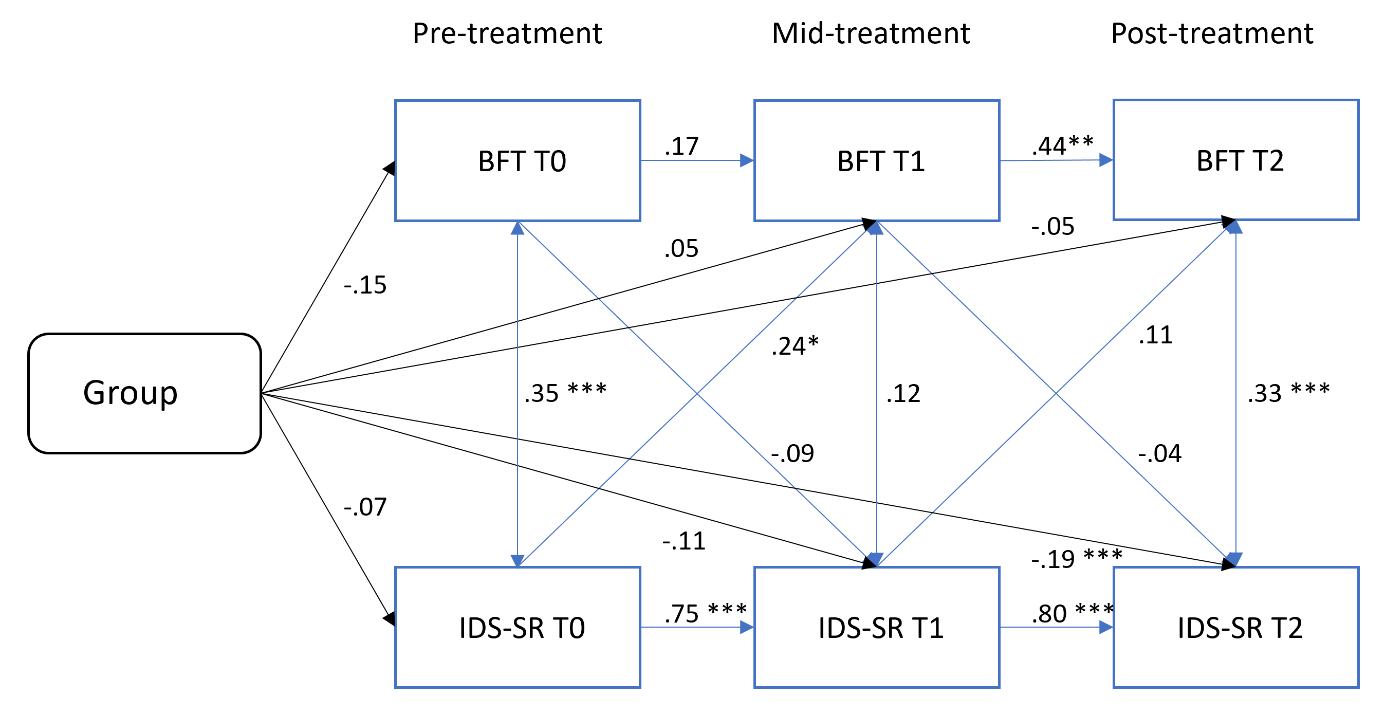


Supplementary Figure S6: Cross-lagged structural equation panel model for mediation of depressive symptoms at post-treatment (T2) by negative intrusive thoughts on the Breathing Focus Task at mid-treatment (T1). Group = Mindfulness-Based Cognitive Therapy (MBCT) + Treatment as Usual (TAU) versus TAU, IDS-SR = Inventory of Depressive Symptomatology – Self-report, BFT = negative intrusive thoughts reported on the Breathing Focus Task.


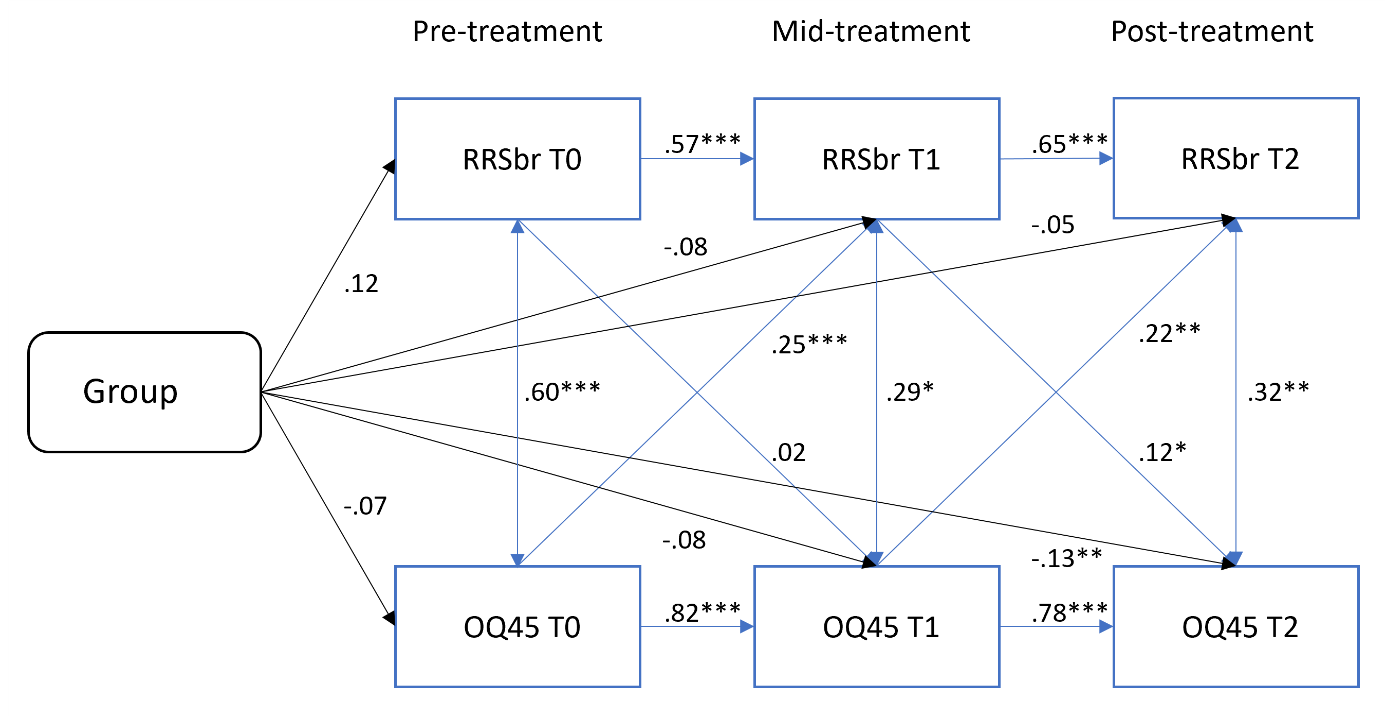


Supplementary Figure S7: Cross-lagged structural equation panel model for mediation of overall functional impairment at post-treatment (T2) by rumination (T1). Group = Mindfulness-Based Cognitive Therapy (MBCT) + Treatment as Usual (TAU) versus TAU, OQ45 = overall functional impairment as measured with the Outcome Questionnaire – 45, RRSbr = Brooding subscale of the Ruminative Response Scale.


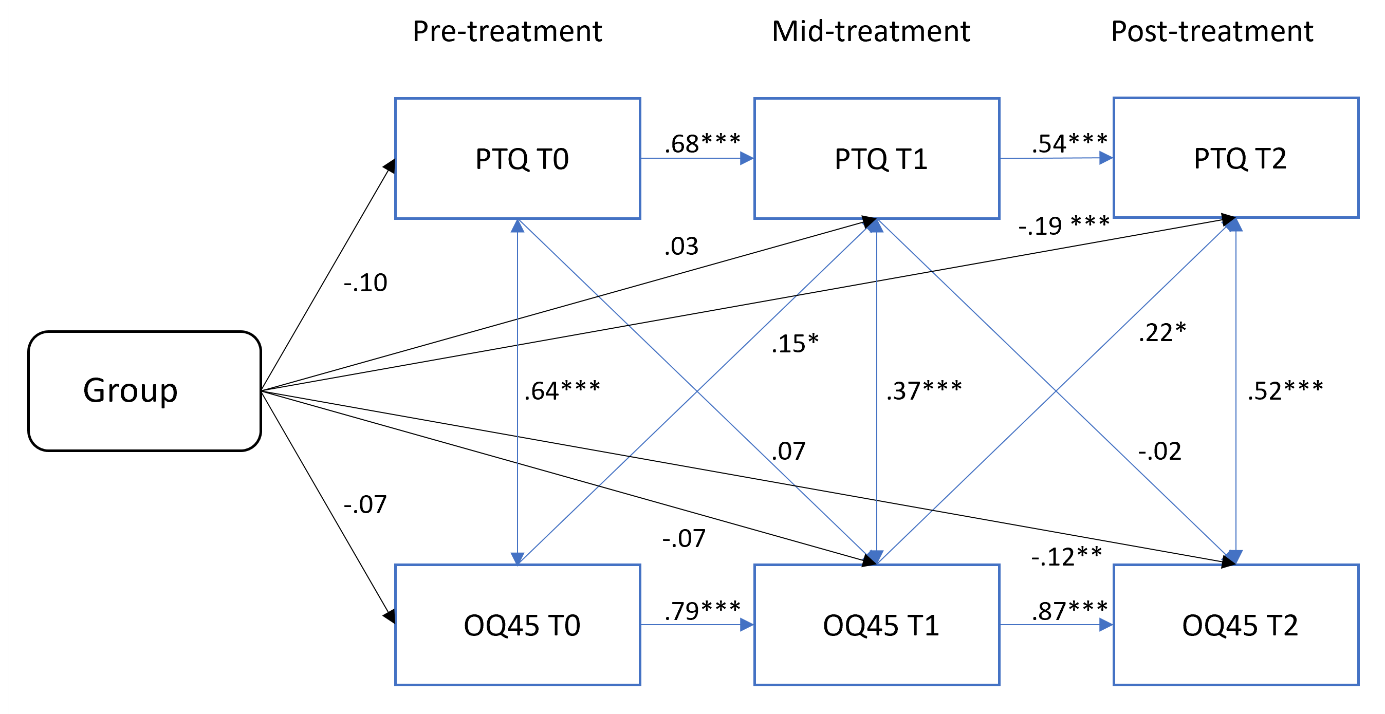


Supplementary Figure S8: Cross-lagged structural equation panel model for mediation of overall functional impairment at post-treatment (T2) by perseverative thinking (T1). Group = Mindfulness-Based Cognitive Therapy (MBCT) + Treatment as Usual (TAU) versus TAU, OQ45 = overall functional impairment as measured with the Outcome Questionnaire – 45, PTQ = Perseverative Thinking Questionnaire.


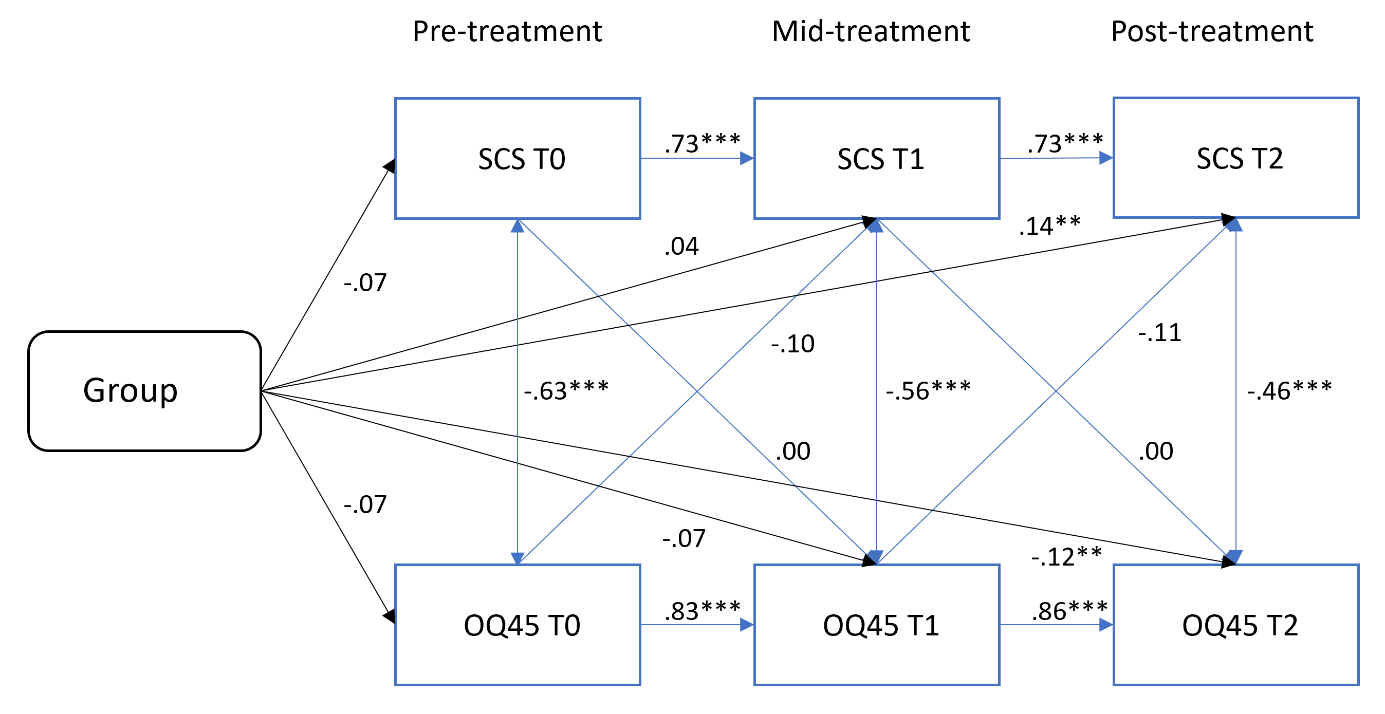


Supplementary Figure S9: Cross-lagged structural equation panel model for mediation of overall functional impairment at post-treatment (T2) by self-compassion (T1). Group = Mindfulness-Based Cognitive Therapy (MBCT) + Treatment as Usual (TAU) versus TAU, OQ45 = overall functional impairment as measured with the Outcome Questionnaire – 45, SCS = Self-compassion Scale – Short-form.


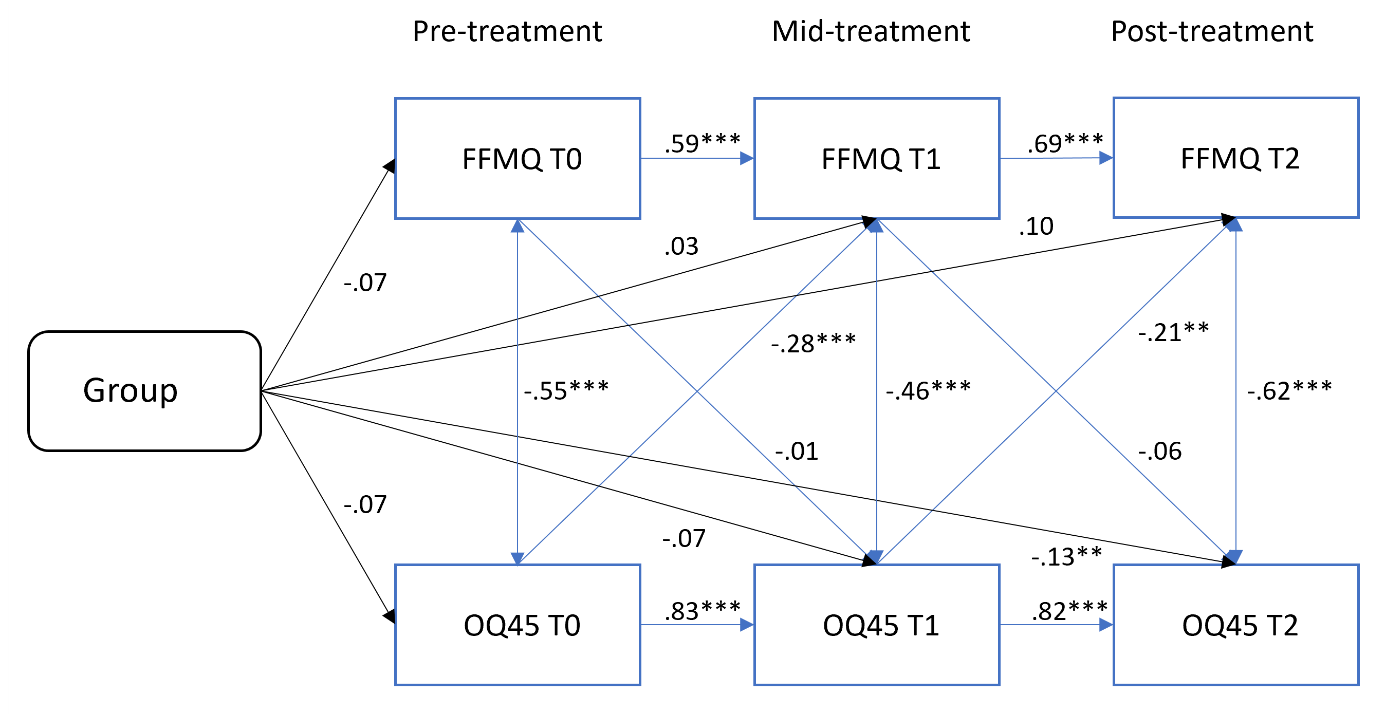


Supplementary Figure S10: Cross-lagged structural equation panel model for mediation of overall functional impairment at post-treatment (T2) by mindfulness skills (T1). Group = Mindfulness-Based Cognitive Therapy (MBCT) + Treatment as Usual (TAU) versus TAU, OQ45 = overall functional impairment as measured with the Outcome Questionnaire – 45, FFMQ = Five Facet Mindfulness Questionnaire – Short-form.


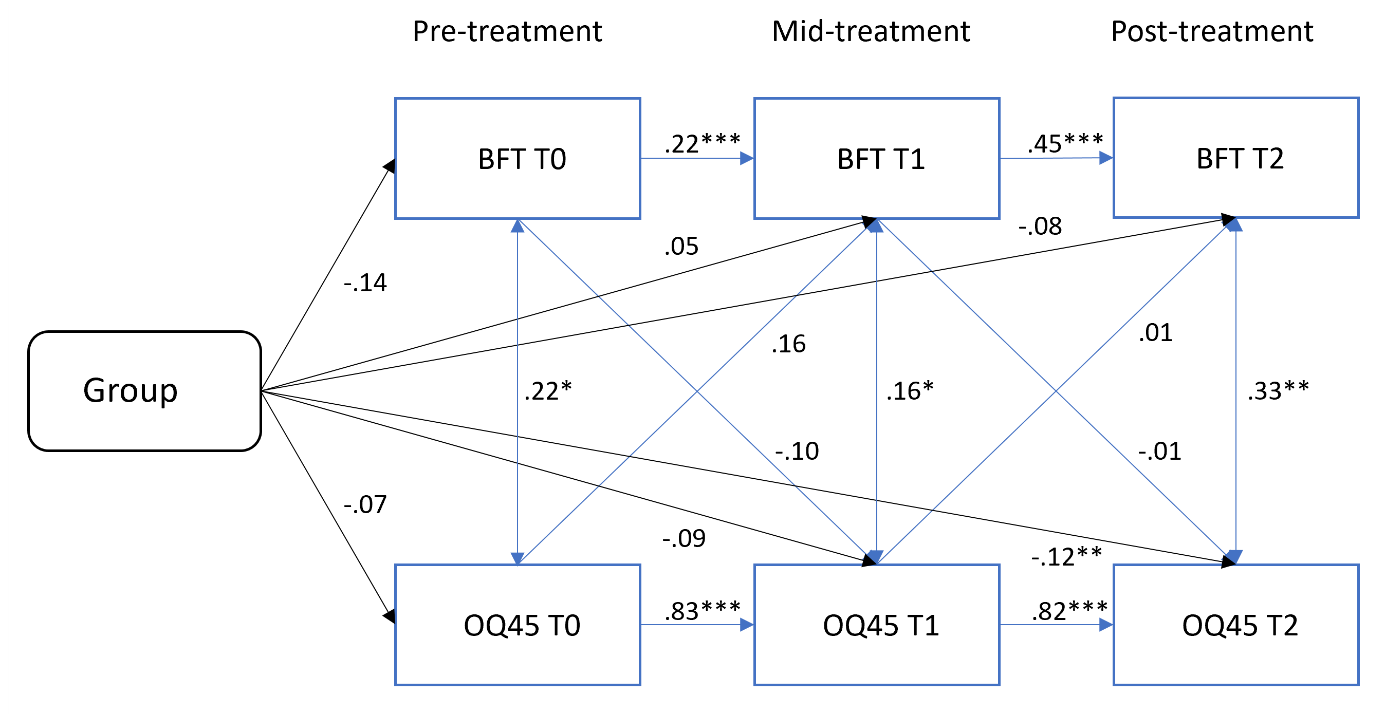


Supplementary Figure S11: Cross-lagged structural equation panel model for mediation of overall functional impairment at post-treatment (T2) by negative intrusive thoughts as reported on the Breathing Focus Task (T1). Group = Mindfulness-Based Cognitive Therapy (MBCT) + Treatment as Usual (TAU) versus TAU, OQ45 = overall functional impairment as measured with the Outcome Questionnaire – 45, BFT = negative intrusive thoughts reported on the Breathing Focus Tasks.
